# High-Density Mapping of Quantitative Trait Loci Controlling Agronomically Important Traits in Quinoa (*Chenopodium quinoa* Willd.)

**DOI:** 10.3389/fpls.2022.916067

**Published:** 2022-06-09

**Authors:** Nathaly Maldonado-Taipe, Federico Barbier, Karl Schmid, Christian Jung, Nazgol Emrani

**Affiliations:** ^1^Plant Breeding Institute, Christian-Albrechts-University of Kiel, Kiel, Germany; ^2^Institute of Plant Breeding, Seed Science and Population Genetics, University of Hohenheim, Stuttgart, Germany

**Keywords:** quantitative trait loci, low-depth sequencing, high-density genetic linkage map, biparental mapping population, phenotypic variation

## Abstract

Quinoa is a pseudocereal originating from the Andean regions. Despite quinoa’s long cultivation history, genetic analysis of this crop is still in its infancy. We aimed to localize quantitative trait loci (QTL) contributing to the phenotypic variation of agronomically important traits. We crossed the Chilean accession PI-614889 and the Peruvian accession CHEN-109, which depicted significant differences in days to flowering, days to maturity, plant height, panicle length, and thousand kernel weight (TKW), saponin content, and mildew susceptibility. We observed sizeable phenotypic variation across F_2_ plants and F_3_ families grown in the greenhouse and the field, respectively. We used Skim-seq to genotype the F_2_ population and constructed a high-density genetic map with 133,923 single nucleotide polymorphism (SNPs). Fifteen QTL were found for ten traits. Two significant QTL, common in F_2_ and F_3_ generations, depicted pleiotropy for days to flowering, plant height, and TKW. The pleiotropic QTL harbored several putative candidate genes involved in photoperiod response and flowering time regulation. This study presents the first high-density genetic map of quinoa that incorporates QTL for several important agronomical traits. The pleiotropic loci can facilitate marker-assisted selection in quinoa breeding programs.

## Introduction

Quinoa (*Chenopodium quinoa* Willd.) is a pseudocereal native to the Andean region of South America. It is an allotetraploid species (2n = 4x = 36), with a genome size of 1.45–1.50 Gb (Jarvis et al., 2017). Quinoa is characterized by its broad genetic variation and adaptation to biotic and abiotic stresses. It exhibits resistance to insects and diseases and tolerance to frost, drought, and salinity. Furthermore, quinoa seeds have outstanding physicochemical, nutritional, and functional properties for human consumption. They have high protein content and are gluten-free. Lysine and eight of the other essential amino acids are present in quinoa seeds in balanced amounts (Melini and Melini, 2021). This crop is considered “functional food” because it contributes to human nutrition while lowering the risk of heart, kidney, and liver diseases (Ali, 2019). The physicochemical properties of quinoa seeds allow the manufacture of processed food, such as puffed quinoa, noodles, and ready-to-eat products (Angeli et al., 2020). Due to these unique qualities, quinoa is considered an option to improve world food security (Alandia et al., 2021).

Quinoa cultivation has transcended continental boundaries and it is present in Europe, Africa, and Asia (Alandia et al., 2020). However, substantial breeding efforts are still needed to explore all quinoa qualities and to expand its cultivation worldwide. Quinoa breeding aims for short, non-branching plants with a compact panicle, as well as increased tolerance to abiotic and biotic stresses. Nevertheless, the main breeding objective in quinoa remains to be the development of high-yielding varieties and, in temperate regions and high latitudes of Europe, North America, and China, the adaptation to long-day conditions (Murphy et al., 2018; Patiranage et al., 2021). Thus, for breeding quinoa, a better understanding of the molecular regulation of flowering time and day-length responsiveness is essential since yield potential and local adaptation are largely determined by these processes.

In spite of being a domesticated crop, quinoa has not yet reached its full potential but molecular and genetic technologies may help change this situation (Alandia et al., 2021). In this scenario, quantitative trait loci (QTL) mapping is useful to understand the genetic basis of quantitative traits. The use of sequencing technologies and computational analysis has made QTL detection easier. In skim sequencing (Skim-seq), genomes are sequenced at low coverage and sequence variants are called after mapping to a reference genome. Later, imputation is performed based on genetic linkage. Due to the large size of linkage blocks, Skim-seq is a suitable method for genotyping F_2_ and F_3_ segregating populations (Golicz et al., 2015; Kumar et al., 2021).

To date, only a few *C. quinoa* linkage maps are available. The first quinoa linkage map was constructed using 216 SSR (simple sequence repeats) markers using a recombinant inbred line (RIL) population. The map consisted of 38 linkage groups (LGs) covering 913 cM (Jarvis et al., 2008). Another linkage map contained 14,178 single nucleotide polymorphism (SNPs) (KASPar genotyping) mapped in two RIL populations. This map consisted of 29 LGs spanning 1,404 cM (Maughan et al., 2012). A recent linkage map by Jarvis et al. (2017) combined the map from Maughan et al. (2012) with two new linkage maps. The resulting map contains 6,403 markers on 18 LGs spanning 2,034 centimorgans (cM). A few studies have attempted to identify loci for agronomically important traits in quinoa so far. Cervantes and van Loo (2017) identified QTL for color, flowering time, and yield-related traits using an F_2_ population of 94 individuals from a cross between “Carina Red” (bitter, dark seed) and “Atlas” (non-bitter EU variety). They used a linkage map constructed with 1,076 SNPs and localized two major QTL, one for days to floral bud appearance on chromosome Cq6B, and another one for seed characters on chromosome Cq2B. In addition, a recent genome-wide association study with 2.9 million markers uncovered significant marker-trait associations for days to flowering, days to maturity, plant height, and panicle length on chromosome Cq2A (Patirange et al., 2020).

In this study, we aimed to create a high-density linkage map and localize QTL for agronomically important traits. A high-density linkage map was constructed with an F_2_ population from a cross between a Chilean and a Peruvian accession. Agronomic traits were assessed in the F_2_ population and the F_3_ generation derived thereof. We mapped a number of highly significant QTL and we identified candidate genes within the QTL confidence intervals. Molecular markers tightly linked to the QTL can be helpful for marker-assisted selection in quinoa breeding programs.

## Materials and Methods

### Plant Material and Growth Conditions

The Chilean quinoa accession PI-614889 (female parent; seed code 171115) was crossed with the Peruvian inbred line CHEN-109 (male parent, seed code 170876) by applying hot water emasculation (Emrani et al., 2020). The F_1_ plant was selfed to give rise to the F_2_ population (seed code: 190031). The F_3_ population (seed codes: 191203-191562) consisting of 334 families, was produced by selfing F_2_ plants (Supplementary Table 1). A total number of 336 F_2_ individuals and 10 plants of each parent were grown in square pots (13 × 13 × 13 cm^3^) from March to October 2019 in a greenhouse under long-day conditions in Kiel, Germany (Supplementary Figure 1). Seeds were harvested from August to October 2019. Three hundred thirty-four F_3_ families and their parental lines were mechanically sown in a plant-to-row scheme in the field in 2020 (10.0°E 54.3°N, Achterwehr, Germany). One hundred fifty seeds were sown in two-meter single rows (1 cm sowing depth) with 50 cm spacing between rows under a complete randomized block design with two blocks. Mechanical weeding was carried out 4 weeks after sowing using a row crop cultivator and hand weeding was performed 5 and 7 weeks after sowing. Thinning was performed 6 weeks after sowing, aiming at 20 plants per row distanced at 10 cm.

### Phenotypic Evaluation

The following traits were assessed in both populations: days to flowering (DTF), days to maturity (DTM), plant height (PH), panicle length (PL), panicle density (PD), saponin content (SC), and thousand kernel weight (TKW). We followed the protocols described by Jarvis et al. (2017) for saponin measurement and those described by Stanschewski et al. (2021) for the other traits with minor modifications as described in Supplementary Table 2. Mildew susceptibility (MS) was recorded only in the F_3_ population in the field, while seed weight per plant (SW) and seed number per plant (SN) were recorded only in the F_2_ population. In the F_2_ trial, 336 individual plants and 10 plants of each parental line were phenotyped. In the field, 10 plants per block and family and the parental lines were phenotyped (a total of 6,720 plants). Plants with significant biotic stress damage in the field (e.g., insect damage) were excluded from phenotyping (Supplementary Table 1). We carried out phenotyping at different Biologische Bundesanstalt, Bundessortenamt, and Chemical Industry (BBCH) stages, which are one of the most widespread scales used to identify the phenological development stages of a plant and were defined for quinoa by Sosa-Zuniga et al. (2017). Additionally, to verify genetic segregation in the F_2_ generation, we phenotyped red axil pigmentation in all 336 individuals.

### Heritability Estimates and Statistical Analysis

The phenotypic (V_*P*_), genotypic (V_*G*_), environmental variances (V_*E*_), and the broad sense heritabilities (h^2^) were estimated using F_3_ data (Falconer, 1996). The heritability values were classified as low (below 30%), medium (30–60%), and high (above 60%) as suggested by Johnson et al. (1955). Genotypic coefficients of variation (GCV), phenotypic coefficients of variation (PCV), environmental coefficients of variation (ECV), and genetic advance with a selection intensity of 5% (GA) were calculated as described by Singh and Chaudhary (1977). In addition, phenotypic correlation coefficients (Pearson’s r) of quantitative traits within and between the F_2_ and F_3_ populations were estimated using the phenotypic value of each F_2_ plant and the average value of each F_3_ family.

### DNA Isolation and Polymerase Chain Reaction

In order to verify genetic segregation by molecular marker analysis, leaf genomic DNA was isolated from 48 F_2_ plants and 194 F_3_ plants by the standard CTAB method (Porebski et al., 1997). We used the InDel marker JASS5 (Fw: AGCCATTG CACTATGCCCTCTC; Rv: TGGCCCAACACCTAAGTGACG) (Zhang et al., 2017). Polymerase chain reaction (PCR) and agarose gel electrophoresis were carried out following the details presented in Supplementary Table 3.

For whole-genome sequencing, we sampled young leaves from 336 F_2_ plants at BBCH 22 and freeze-dried them. We extracted DNA from these samples by a modified protocol of the Genomic Micro AX Blood Gravity kit (A&A Biotechnology, Gdansk, Poland). We verified the quality of the isolated DNA by agarose gel electrophoresis (0.8%, 60 V, 60 min).

### Whole-Genome Sequencing and Bioinformatics

Whole-genome sequencing libraries were constructed using the protocol of Baym et al. (2015) and normalized for equimolarity using a BioTec Synergy HTC multimode plate reader. The library was sequenced by Illumina NovaSeq PE150. We aimed to ∼1× coverage per sample of whole-genome sequences of the F_2_ individuals (Skim-seq). The genome sequences of both parents, CHEN-109 and PI-614889, were already available with a coverage of 7.45× and 8.00×, respectively (Patirange et al., 2020). We trimmed raw reads with Trim_galore v 0.6.4 (parameters -q 30 –fastqc –paired) (Krueger, 2015), sorted and indexed them with SAMTOOLS 1.10 (Li et al., 2009), and deduplicated them with MarkDuplicates (parameter REMOVE_DUPLICATES = TRUE tool of PICARD v2.21.9) (Broad-Institute, 2019). Quality control was done with FastQC (v0.11.9) and MultiQC (v1.9) (Ewels et al., 2016) by removing reads containing *N* > 10% (*N* represents the percentage of the nucleotides that cannot be determined) and a quality base filter of Qscore = 5 (over 50% of the total base). We mapped the reads to the Quinoa Reference Genome QQ74_V2 (CoGe Genome ID: id60716). We called variants using HaplotypeCaller (v4.1.8.1) in -ERC GVCF mode (Poplin et al., 2017). Markers were named as “chromosome number_physical position” (e.g., chr12_ 2345937). We kept only homozygous loci within each parent and considered only SNPs with a minimum base quality of 30 (minQ = 30) and minor allele frequency (maf): 0.1. Then, we imputed the missing data by FSFHap from TASSEL (v.5.2.64) (maf: 0.1 MaxMissing: 0.8; Window: 50) (Swarts et al., 2014). To verify imputation accuracy, we generated data sets in which SNPs with a minimum read depth of eight (minDP = 8) were masked. We generated six data sets, one per chromosome: Cq1A, Cq1B, Cq2A, Cq2B, Cq3A, and Cq3B. Then, we imputed the masked data sets using FSFHap with the same parameters as described previously. We evaluated the imputation accuracy by genotype concordance between the masked-and-imputed SNPs and the original genotypes. Genotype concordance was evaluated by SnpSift (v.5.1) (Ruden et al., 2012) and reported as percentage of similarities between masked and original genotypes.

After imputation, we applied the following filters: min-alleles: 2; max-alleles: 2 max-missing: 0.3; maf: 0.1. Finally, we transformed the data to a parent-based format (.abh) by using the GenosToABH plugin from TASSEL (v.5.2.64), using the codes A: male parent, B: female parent, H: heterozygous. We performed quality control of the imputed data in.abh format by segregation distortion and percentage of missing data (ABHgenotypeR v.1.0.1 R package) (Furuta et al., 2017). The bioinformatics pipeline is illustrated in Supplementary Figure 2A.

### Linkage Map Construction

First, we performed final filtering of the F_2_ population (Supplementary Figure 2B). We excluded F_2_ plants with more than 30% missing data. Only markers present in more than 302 F_2_ plants and fitting a 1:2:1 (α = 0.05) segregation ratio were used for linkage studies. We also excluded “identical” individuals with >95% sequence similarity. Then, we constructed the genetic map by MSTMap (Wu et al., 2008) with the following parameters: Kosambi function, cut-off *p*-value = 1e-09, no_map_dist: 15, no_map_size: 2, missing_threshold: 0.1. Markers with an estimated genetic distance ≤ 1.00E-04 cM were clustered into bins. Finally, we performed several analyses for quality control of our genetic map. To begin with, we checked segregation distortion and estimated the number of crossovers and double-crossover following the guidelines given by Broman (2010). In addition, we analyzed parental allele frequencies and collinearity of our linkage map with the physical map. Moreover, we used a heatmap of our linkage map to look for switched alleles. We used LinkageMapViewR (v.2.1.2), ASMapR (v.1.0-4), and R/qtl (v.1.46-2) for quality control of the genetic map (Broman et al., 2003; Taylor and Butler, 2017; Ouellette et al., 2018).

### Quantitative Trait Loci Mapping and Pleiotropy Analysis

We carried out QTL mapping by composite interval mapping using the software package R/qtl (v.1.46-2) (Haley-Knott with forward selection to three markers and a window size of 10 cM) (Broman and Sen, 2009). The threshold for the logarithm of odds (LOD) for a significant QTL declaration was calculated by 1,000 permutations of the genome-wide maximum LOD. The 95th and 99th percentile of this distribution were used as the genome-wide LOD thresholds (5 and 1% LOD thresholds). The confidence intervals were calculated using the 95% Bayes credible interval method. QTL effects were calculated with the nearest markers as the phenotypic differences between marker genotypes. The percentage of phenotypic variation explained by each QTL (R^2^) was estimated by “drop-one-QTL-at-a-time” analysis. A simple additive model for multiple QTL was generated for each trait using the multiple imputation method and the Haley-Knott regression. When a putative pleiotropy was observed, it was confirmed by the qtlpvl R package (v.0.1-2), and a multiple trait QTL analysis was performed (Tian et al., 2016). After confirmation of pleiotropy, pleiotropic sites were analyzed as single multitrait QTL (scanone.mvn) to obtain Bayes intervals and R^2^ values.

### Epistasis Analysis

A genome-wide epistasis analysis was performed to describe how alleles influence each other. For this purpose, we used the cape R package (v.3.1.0) (Tyler et al., 2013). To facilitate the analysis in terms of computational time, the software decomposed the phenotypes matrix into eigentraits (ET) by singular value decomposition (SVD). Then, we selected the two ET capturing the highest total variance among the traits to perform a pairwise scan of the variants (SNPs). From this scan, the software found interactions between alleles (epistasis), and the epistatic models were combined across ET to find allelic effects on the phenotypes included in the ET. Positive and negative allelic effects refer to the reparametrized coefficient (either < 0 or > 0) from the pairwise regression as described by Tyler et al. (2013). Ultimately, the results of this analysis describe how alleles influence each other, in terms of enhancement (positive coefficient) and suppression (negative coefficient), as well as how gene variants influence phenotypes. The results of this analysis were plotted as heatmaps. ET contribution to the phenotypes was estimated for all bins of our genetic map, and heatmaps were constructed with 1,000 randomly selected markers. Effect calculations were performed in reference to the female parent (PI-614889) allele.

### Candidate Gene Identification and Haplotype Analyses

We retrieved annotated genes from the reference genome within the regions of the confidence intervals of each QTL to explore possible candidate genes (.gff from QQ74_V2; CoGe Genome ID: id60716). We selected preliminary candidate genes using the UniProt Knowledgebase database (UniProtKB). A gene was considered a candidate when a related function to the identified QTL was already described in other plant species. Then, we searched for variants (SNPs and InDels) within the parental sequences. From this search, we kept homozygous genes and gave preference to those variants with a putative effect on the function of the encoded protein. Following, we evaluated the haplotype of the selected variants in the F_2_ population, as follows: we clustered the phenotypes according to the corresponding genotype at the variant site; later, we performed t-tests (α = 0.05) to compare DTF, PH, and TKW among the created clusters. To further evaluate the phenotypic effect of the variants, we used whole-genome sequencing and phenotypic data of 310 quinoa accessions grown in a 2-year experiment in Kiel, Germany. This dataset comprises 2.9 million high confidence SNP and 414,891 InDel loci (Patirange et al., 2020). We followed the same procedure as for the haplotype evaluation in the F_2_ population. We assigned letters for each allele to describe the genotypes (e.g., *A_1_A_1_* homozygous, *A_1_A_2_* heterozygous). A complete description of the nomenclature is given in Supplementary Table 4.

## Results

### Segregation and Phenotypic Analysis of F_2_ and F_3_ Populations

We verified the expected 1:2:1 genetic segregation in the F_2_ population by two approaches: phenotyping of the red axil pigmentation (complete dominance of red color over green color) (Simmonds, 1971) and molecular marker analysis. We phenotyped red axil pigmentation in all 336 F_2_ individuals while genotyping was carried out for 48 individuals. Likewise, the expected segregation in the F_3_ generation (3:2:3) was verified by molecular markers. One hundred ninety-four plants were genotyped from 20 randomly selected F_3_ families (Supplementary Table 5 and Supplementary Figure 3).

Both populations, F_2_ and F_3_, exhibited a vast phenotypic variation under field and greenhouse conditions (Supplementary Figure 4). Moreover, substantial transgressive segregation was found for all traits (Table 1). The highest transgression percentage was found for TKW in the F_2_ generation. On the other hand, heritabilities ranged between 38.02 and 91.06% with TKW exhibiting the highest heritability value (91.06%). Besides, DTF, DTM, PD, and MS showed high heritability (79.77–82.99%) while PH and PL exhibited moderate heritability (38.44 and 38.02%, respectively) (Table 2). Importantly, only 34.9% of the plants reached maturity before harvesting in the field (October 2020), resulting in fewer plants being phenotyped for DTM in the F_3_ population (Supplementary Table 1).

**TABLE 1:** Results from the quinoa populations grown in the greenhouse (F_2_) and the field (F_3_) and their parental lines.

Character	Population/parent	Mean	Minimum	Maximum	Transgression (%)
DTF	CHEN-109	2019: 61 ± 2^a^	−	−	−
		2020: 72 ± 3^a^			
	PI-614889	2019: 76 ± 1^b^	−	−	−
		2020: 96 ± 4^b^			
	F_2_	69 ± 5	55	87	29.17
	F_3_	82 ± 9	67	77	14.63
DTM	CHEN-109	2019: 177 ± 7^b^	−	−	−
		2020: NA			
	PI-614889	2019: 110 ± 2^a^	−	−	−
		2020: 129 ± 4			
	F_2_	137 ± 14	110	194	11.91
	F_3_	152 ± 9	123	165	NA
PH (cm)	CHEN-109	2019: 151.40 ± 7.06^b^	−	−	−
		2020: 164.74 ± 27.56^b^			
	PI-614889	2019: 83.90 ± 6.00^a^	−	−	−
		2020: 137.11 ± 13.98^a^			
	F_2_	136.3 ± 20.63	83.00	193.00	22.02
	F_3_	163.14 ± 27.85	50.00	265.00	66.49
PL (cm)	CHEN-109	2019: 37.20 ± 4.32^b^	−	−	−
		2020: 32.37 ± 6.74^a^			
	PI-614889	2019: 16.30 ± 2.49^a^	−	−	−
		2020: 29.47 ± 9.41^a^			
	F_2_	24.07 ± 6.14	14.00	50.00	8.33
	F_3_	32.85 ± 10.4	10.00	80.00	76.31
PD	CHEN-109	2019: 3.00 ± 0.00^a^	−	−	−
		2020: 1.05 ± 0.23^a^			
	PI-614889	2019: 5.00 ± 0.00^b^	−	−	−
		2020: 2.68 ± 0.58^b^			
	F_2_	3.61 ± 1.49	1	7	14.88
	F_3_	2.49 ± 1.05	1	5	77.17
TKW (g)	CHEN-109	2019: 2.00 ± 0.14^b^	−	−	−
		2020: 1.52 ± 0.06^b^			
	PI-614889	2019: 2.44 ± 0.31^a^	−	−	−
		2020: 2.64 ± 0.03^a^			
	F_2_	2.75 ± 0.49	1.18	3.91	77.97
	F_3_	2.62 ± 0.29	1.58	3.45	48.17
SW (g)	CHEN-109	2019: 1.07 ± 0.26^b^	−	−	−
	PI-614889	2019: 5.90 ± 0.88^a^	−	−	−
	F_2_	4.10 ± 1.48	0.06	7.22	13.29
SN	CHEN-109	2019: 633 ± 161^b^	−	−	−
	PI-614889	2019: 2,432 ± 328^a^	−	−	−
	F_2_	1469 ± 471	51	3,251	6.25
SC	CHEN-109	2019: 6.84 ± 0.20^b^	−	−	−
		2020: 6.5 ± 2.12^b^			
	PI-614889	2019: 3.20 ± 1.09^a^	−	−	−
		2020: 2.00 ± 0.00^a^			
	F_2_	5.68 ± 4.22	2.00	24.00	47.02
	F_3_	12.61 ± 8.28	2.00	30.00	57.97
MS	CHEN-109	2020: 2.95 ± 0.23^b^	−	−	−
	PI-614889	2020: 2 ± 0.64^a^	−	−	−
	F_3_	2.34 ± 0.69	1	3	59.42

*DTF, days to flowering; DTM, days to maturity; PH, plant height; PL, panicle length; PD, panicle density; TKW, thousand kernel weight; SW, seed weight per plant; SN, seed number per plant; SC, Saponin content; MS, Mildew susceptibility. NA, data not available due to late-maturity genotype. Different letters ^a,b^Indicate significant differences between parental lines in each year (F_2_:2019; F_3_:2020) (t-test, α = 0.05).*

**TABLE 2:** Statistical parameters calculated for eight phenotypic characters measured in the F_3_ population.

Parameter	DTF	DTM	PH	PL	PD	MS	TKW	SC
V_P_	72.54	81.01	775.68	108.15	1.10	0.48	0.02	8.28
V_G_	60.20	64.62	298.20	41.11	0.90	0.40	0.02	7.22
V_E_	12.34	16.39	477.49	67.03	0.20	0.08	0.002	1.06
PCV (%)	10.34	5.94	17.07	31.66	42.06	41.67	5.70	22.81
GCV (%)	9.42	5.30	10.59	19.52	38.12	37.93	5.44	21.30
ECV (%)	4.26	2.67	13.39	24.93	17.78	17.26	1.70	8.16
h^2^ (%)	82.99	79.77	38.44	38.02	82.14	82.85	91.06	87.19
GA (%)	17.68	9.76	13.52	24.79	71.16	71.12	10.70	40.97

*V_P_, phenotypic variance; V_G_, genotypic variance; V_E_, environmental variance; PCV, phenotypic coefficient of variation; GCV, genotypic coefficient of variation; ECV, environmental coefficient of variation; h^2^, broad-sense heritability; GA, genetic advance; DTF, days to flowering; DTM, days to maturity; PH, plant height; PL, panicle length; PD, panicle density; TKW, thousand kernel weight; SC, saponin content; MS, Mildew susceptibility.*

**FIGURE 1:**
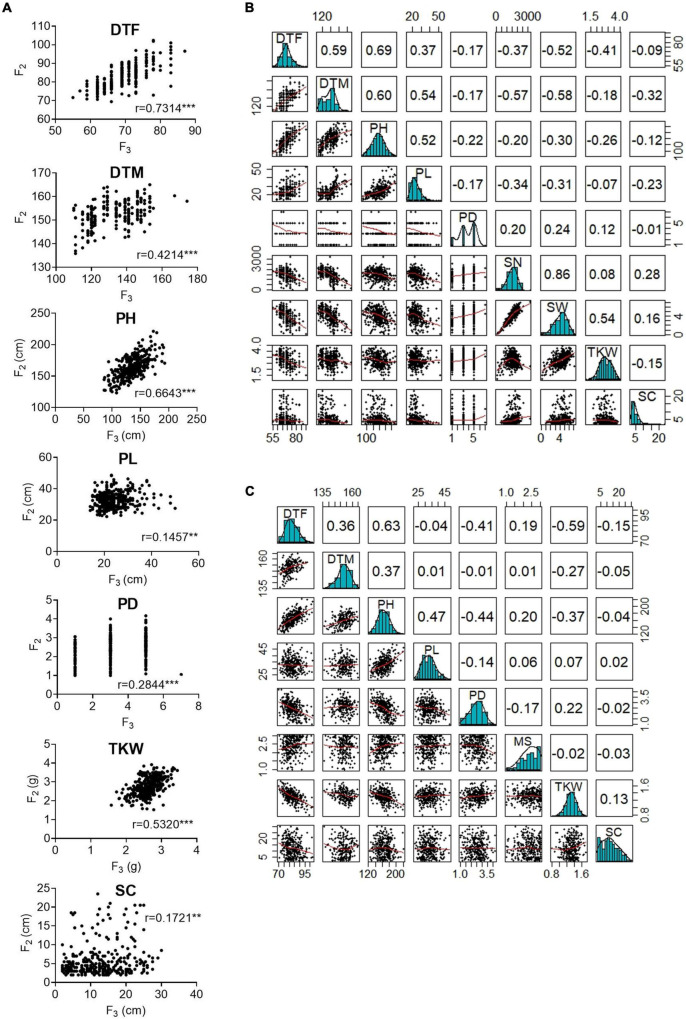
Correlation between phenotypic traits measured in the F_2_ and F_3_ populations. **(A)** Pearson’s r correlations between F_2_ and F_3_ populations. **(B)** Correlations between nine traits measured in the F_2_ population **(C)** Correlations between eight traits measured in the F_3_ population. In panels **(B,C)**, bivariate scatter plots are shown below the diagonal, histograms on the diagonal, and the Pearson correlation above the diagonal. DTF, days to flowering; DTM, days to maturity; PH, plant height; PL, panicle length; PD, panicle density; TKW, thousand kernel weight; SW, seed weight per plant; SN, seed number per plant; MS, Mildew susceptibility; SC, saponin content. Correlations significance at α< 0.05 = ***, α< 0.01 = **, α< 0.001 = * levels.

Then, we calculated correlations between all evaluated traits within years. The highest correlation was found between DTF and PH (Pearson’s r in F_2_: 0.69; Pearson’s r in F_3_: 0.63) (Figure 1). Both traits, PH and DTF, were significantly correlated with DTM. Furthermore, DTF showed a high negative correlation with TKW (F_2_ and F_3_) and with SN and SW (only F_2_). In general, taller plants flowered later, reached maturity later, and depicted a reduction in the yield traits values, while shorter plants flowered earlier, reached maturity earlier, and showed higher values for the yield-related traits. Additionally, significant correlations for DTF, DTM, PH, PD, and PL between the F_2_ plants grown under greenhouse conditions and their F_3_ progenies were calculated, with the highest values for DTF (0.73) and PH (0.66). Surprisingly, SC showed a low correlation between years (Pearson’s r: 0.17).

### Sequencing the F_2_ Population Revealed Millions of Single Nucleotide Polymorphism

We sequenced the genomes of 336 F_2_ plants, the parents of the F_3_ families grown in the field (accession numbers^1^ : from SRR18906894 to SRR18907229). Skim-Seq by Illumina NovaSeq PE150 resulted in a total data output of 4.98 million raw PE reads on average per individual (∼1.07× coverage). All reads passed the quality base filter requirement (Qscore = 5), and 0.0053% of the raw data were removed due to a high number of nucleotides that could not be determined (*N* > 10%).

**FIGURE 2:**
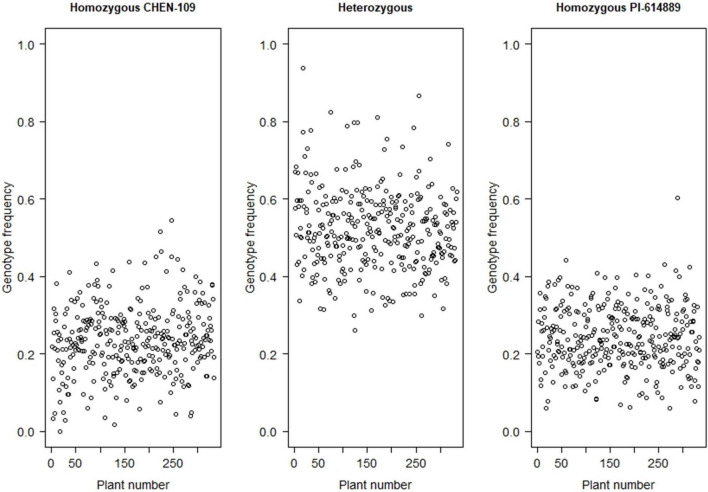
Frequency distribution of the homozygous genotype from the parent CHEN-109, the homozygous genotype from the parent PI-614889, and heterozygous genotype, for each of the F_2_ individuals.

Seventeen million SNPs were obtained after mapping and variant calling (Supplementary Figure 5). First, these SNPs were filtered by maf: 0.1 and minQ30, producing a data set of four million high-quality SNPs with a high percentage of missing data (∼86%) (Supplementary Figure 6). After imputation, the proportion of missing data was reduced from ∼86.0 to ∼11.0%. Imputation accuracy, evaluated by genotype concordance between original and masked-and-imputed genotypes, varied from 99.69 to 99.95% (99.85% in average) (Supplementary Table 6). Following the next filtering steps, we obtained a set of 249,744 high-quality biallelic SNPs with 4.2% missing data, 21.2% homozygous markers for the male parent allele, 26.5% homozygous markers for the female parent allele, and 52.3% heterozygous markers (Figure 2). We used this set of markers for genetic map construction.

### Construction of a High-Density Linkage Map

Ahead of genetic map construction, the F_2_ population sequences were cleaned anew based on our quality criteria. Two plants were removed due to >30% missing data (Supplementary Figure 7A), and 15,933 markers were removed because they were missing from >10% of the population (Supplementary Figure 7B). Another 99,898 markers were removed because they did not segregate in the expected 1:2:1 manner. No plants had to be removed due to high (>95%) sequence similarity to another F_2_ plant (Supplementary Figure 7C). As outcome of our final filtering, 334 F_2_ plants were used to construct a genetic map with 133,913 markers, resulting in an average density of one marker per ∼8.97 Kb. The resulting genetic map consists of 21 linkage groups (LG), with the chromosomes 5B, 6B, and 8B split into two LGs each (Table 3 and Supplementary Figure 7). Moreover, the linkage map has an average density of ∼88 markers per cM, where one cM corresponds to ca. 0.83 Mb (Supplementary Figure 9). For further steps, we created 5,218 bins where the markers with a genetic distance ≤ 1.00E-04 cM were clustered into.

**TABLE 3:** Summary statistics of the quinoa linkage map based on F_2_ plants derived from a cross between CHEN-109 and PI-614889.

Chromosome	Size (Mb)	Linkage group	Size (cM)	No. of markers	No. of bins
Cq1A	57.13	1	82.3	11,679	323
Cq1B	71.68	2	79.9	2,377	180
Cq2A	53.75	3	87.3	11,397	310
Cq2B	73.59	4	110.4	5,637	340
Cq3A	57.38	5	97.5	8,827	349
Cq3B	72.83	6	77.5	8,770	253
Cq4A	59.38	7	114.3	9,473	380
Cq4B	71.74	8	94.3	9,634	360
Cq5A	64.66	9	90.8	10,824	323
Cq5B	78.03	10	22.4	1,162	103
		11	32.3	2,405	120
Cq6A	66.92	12	116.6	5,559	317
Cq6B	87.28	13	24.1	1,923	119
		14	25.3	873	109
Cq7A	57.68	15	51.9	3,879	205
Cq7B	75.96	16	91.2	9,004	273
Cq8A	59.27	17	104.1	12,687	502
Cq8B	75.04	18	7.00	570	42
		19	46.5	1,636	117
Cq9A	54.51	20	83.5	8,551	228
Cq9B	63.80	21	80.9	7,046	265
Total	1,200.71	−	1,520.1	133,913	5,218

*The physical size of each chromosome was taken from the reference genome QQ_74 (CoGe Genome ID: id60716).*

**FIGURE 3:**
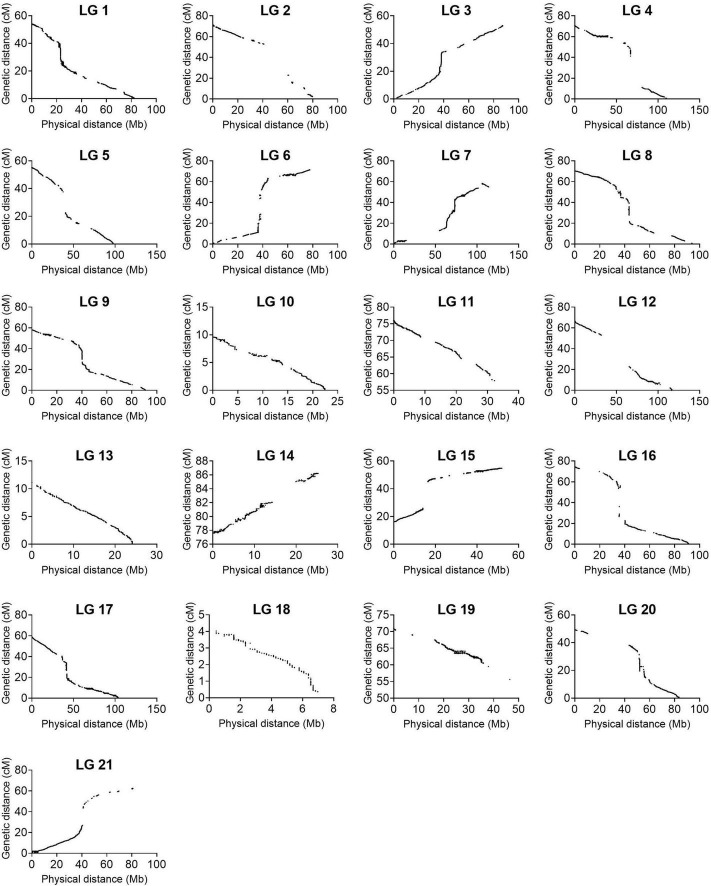
Collinearity between the linkage map constructed in this study and the physical map from the reference genome QQ74_V2 (CoGe Genome ID: id60716). The graphs were constructed with 133,913 non-binned markers.

**FIGURE 4:**
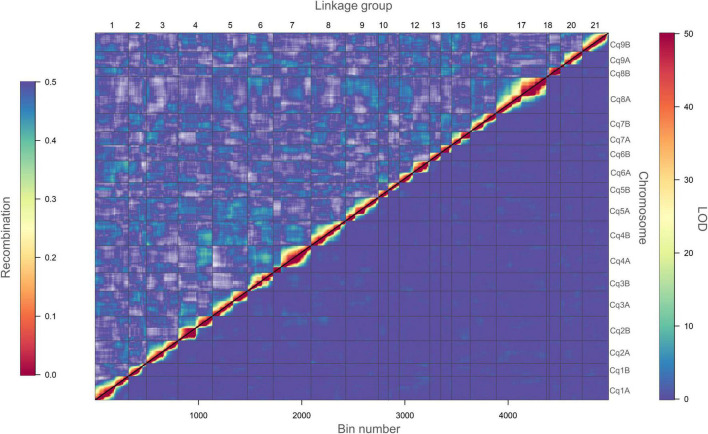
Heatmap of pairwise recombination fractions and LOD scores based on 5,219 bins. Estimated recombination fractions between binned markers are shown above the diagonal and LOD scores below the diagonal. Red colors indicate closely linked binned markers (high LOD score and low recombination fraction), whereas, blue colors indicate non-linked binned markers (low LOD score and high recombination fraction). A LOD score of 50 corresponds to a recombination fraction of zero. Grid lines divide the binned markers by linkage groups (vertically) and by chromosomes (horizontally).

To continue, we carried out several quality control analyses on the genetic map. First, we checked the number of single and double crossovers per plant, which ranged from 10 to 105 and 0 to 9, respectively (Supplementary Figure 10A). We did not find any outlier plants depicting a significantly higher number of crossovers and double crossovers than the ones observed for the population, which would have indicated potential genotyping errors (Supplementary Figure 10B). Second, we analyzed the collinearity of our genetic map with the physical map from the reference genome sequence V2 and observed high collinearity. We observed major gaps at the centromeres (up to ∼33 cM) and an inversion at LG 7 (Figure 3 and Supplementary Figure 8). Third, we investigated switched alleles by a heatmap (Figure 4). We did not find switched alleles, which would be indicated by pairs of markers with low LOD scores and low recombination fractions. Moreover, we inspected the parental allele frequencies in each linkage group, which were as expected: 0.25 for CHEN-109 genotype, 0.25 for PI-614889 genotype, and 0.5 heterozygous genotypes (Supplementary Figure 11).

### Quantitative Trait Loci Mapping, Pleiotropic Loci Identification, and Epistasis Calculation

**FIGURE 5:**
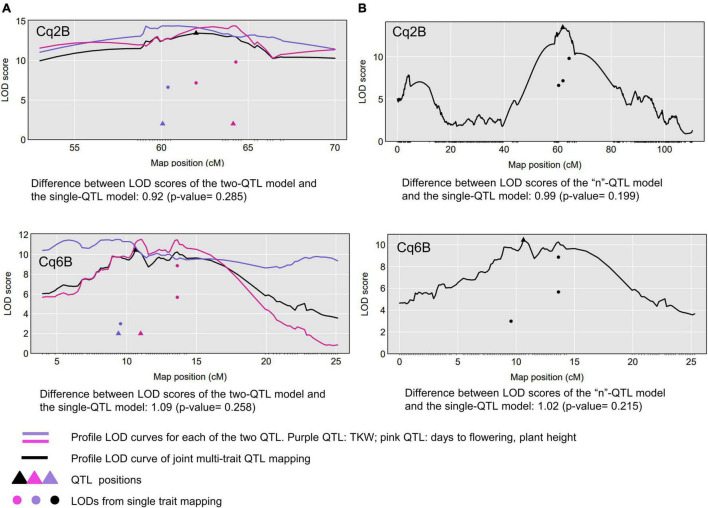
Comparative QTL analysis to detect pleiotropic QTL. Two tests were performed: **(A)** one vs. two QTL and **(B)** one vs. “*n*” QTL. Tests were performed considering the traits involved in the QTL found in common for F_2_ and F_3_ populations (top graphs: *pleio4.1*; bottom graphs: *pleio14.1*). The black curve is the LOD score curve for the single-QTL model, with the estimated QTL location indicated by a black triangle. The blue and pink curves are profile LOD score curves for the for the two-QTL model. Dots indicate the LOD score for the traits considering a single-QTL model.

We mapped QTL for ten agronomically important traits using phenotypic data of 334 F_2_ plants and 328 F_3_ families, which had passed our quality check (Supplementary Data Sheet 2). Fifteen QTL were identified, ranging from one to three QTL per trait (Table 4). We found pleiotropy at seven QTL, which were named with the prefix “*pleio*” (Figure 5 and Supplementary Figure 12). Two QTL (*pleio4.1* and *pleio14.1*) were in common between F_2_ and F_3_, whereas six and eight QTL were found only in F_2_ or F_3_ populations, respectively. Together, *pleio4.1* and *pleio14.1* explained 22.01% of the phenotypic variation for TKW, PH, and DTF, being this the strongest effect observed among all QTL. *pleio20.1* and *pleio4.1* showed the highest additive and dominance effect, respectively.

**TABLE 4:** Summary statistics of quantitative trait loci (QTL) mapping with the F_2_ population and 328 F_3_ families.

Trait	Population	QTL name	Confidence Interval (cM)	Flanking markers	LG	Chr	R^2^ (%)		*p*-value	Effect^a^		No. of genes^b^
							QTL	Trait		Additive	Dominant	
DTF, PH, TKW	F_2_ and F_3_	*pleio4.1*	69.10–64.44	chr4_53552445, chr4_50421644	4	Cq2B	10.55	22.01	3.68E-03	−7.17	8.74	104
		*pleio14.1*	8.95–16.00	chr12_80422064, chr12_82045210	14	Cq6B	6.52		3.68E-03	6.73	−3.17	178
PH, PL, PD	F_3_	*pleio20.1*	10.39–22.00	chr17_46536722, chr17_38090534	20	Cq9A	10.86	10.86	6.47E-09	−9.13	−3.25	660
DTF, PH, PD, TKW		*pleio4.2*	69.00–64.59	chr4_53811433, chr4_50411615	4	Cq2B	12.44	12.44	3.43E-10	−6.79	7.68	110
DTM		*dtm3.1*	35.64–43.00	chr3_17349205, chr3_37087692	3	Cq2A	8.73	8.73	3.42E-05	−0.58	0.32	470
MS		*ms4.1*	1.68–7.00	chr4_69599485, chr4_66031413	4	Cq2B	0.38	0.43	0.264	−0.17	0.11	410
		*ms5.1*	70.00–76.69	chr5_14713288, chr5_9505959	5	Cq3A	0.06		0.955	−0.22	0.10	407
PD		*pd16.1*	42.32–44.72	chr14_18518421, chr14_17111206	16	Cq7B	10.80	20.44	1.99E-15	−0.28	0.03	47
DTF, DTM, PH, PL, SW, SN, TKW	F_2_	*pleio4.3*	59.62–62.00	chr4_53467737, chr4_ 50761666	4	Cq2B	16.21	16.21	1.78E-13	−3.21	0.41	88
DTF, PH, PD, SW		*pleio7.1*	5.32–20.00	chr7_2959770, chr7_3472874	7	Cq4A	10.97	10.97	5.02E-09	−2.31	−1.31	56
SN		*sn6.1*	35.89–37.54	chr6_12791379, chr6_23647528	6	Cq3B	2.77	5.46	0.010	1.02	0.15	313
TKW		*tkw17.1*	18.98–23.78	chr15_48085872, chr15_45144697	17	Cq8A	4.02	8.14	0.001	−0.96	−1.01	156
Saponin		*sap10.1*	0.00–2.63	chr10_9590242, chr10_8685894	10	Cq5B	6.60	21.98	3.68E-03	−1.51	−0.69	80
		*sap13.1*	14.61–20.93	chr12_ 5194791, chr12_ 2345937	13	Cq6B	9.31		3.68E-03	−1.39	−1.68	262
		*sap17.1*	2.25–10.21	chr15_57094405, chr15_52868334	17	Cq8A	6.63		3.68E-03	−1.43	0.60	365

*The trait acronyms are explained in the methods section. LG, linkage group; Chr, chromosome; R^2^, estimated percentage of the phenotypic variance explained by the QTL. ^a^Relative to the PI-614889 allele. ^b^Genes within the confidence interval.*

**FIGURE 6:**
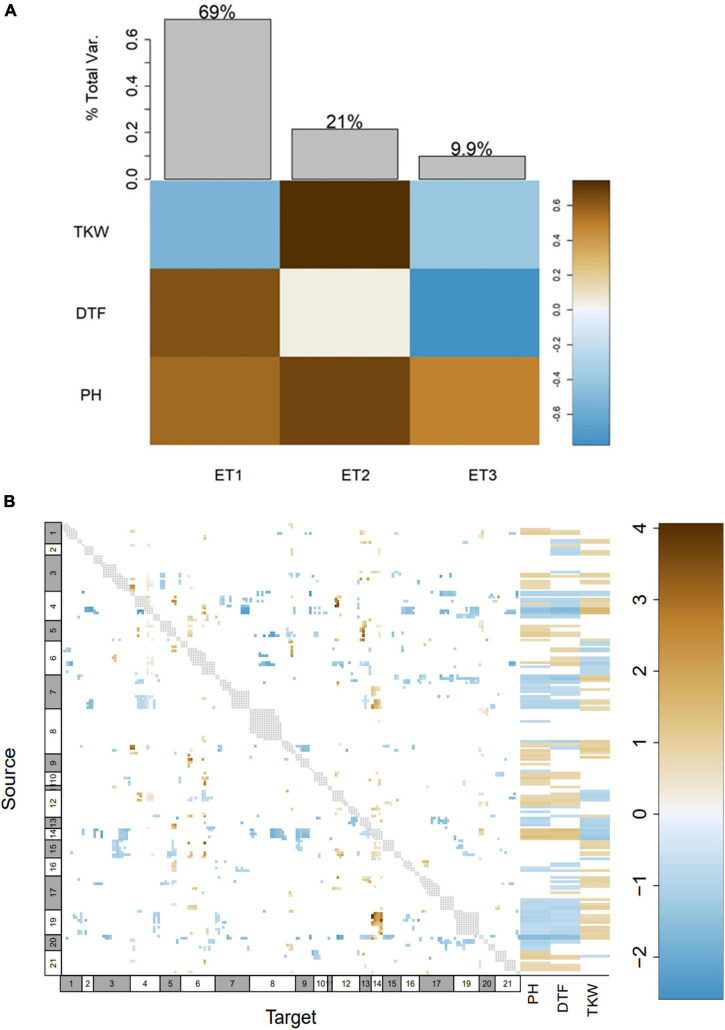
Genome-wide epistasis analysis and effects of alleles at genome level on days to flowering (DTF), plant height (PH), and thousand kernel weight (TKW). **(A)** Decomposition of phenotypes into eigentraits (ET). Colors of the heatmap correspond to the global variance fraction of each ET. **(B)** Heatmap showing interactions between all pairs of alleles at genome level, resulting from a pairscan analysis. Every allele was assigned as “source” and “target” for the pairscan analysis. To the right of the heatmap, the interaction of every allele (assigned as source) on the phenotypes involved in the pleiotropy is shown. The heatmap scale represents the reparametrized coefficient calculated by the software and might be interpreted as the direction of the allelic effect. Gray dots show marker pairs that were not included in the pairwise scan due to complete linkage. Numbers at the x and y axes in white and gray boxes correspond to linkage groups.

We performed a genome-wide epistasis analysis to investigate how alleles influence each other in terms of enhancement and suppression and also examined how different alleles of genes influence phenotypes (DTF, PH, and TKW). As the first step of this epistasis analysis, the phenotype matrix was decomposed by singular value decomposition (SVD) into eigentraits (ET). Two ET captured 69.00 and 21.00% of the total variance among DTF, PH, and TKW and were selected to perform a pairwise scan of the SNPs (Figure 6A). Then, we constructed a heatmap where 46,52% of the alleles at the genome level had a minor simultaneous effect (>-1 or <1) on DTF, PH and TKW. Moreover, alleles located within the pleiotropic region *pleio4.1* showed 252 significant interactions with other alleles in all LGs, except for LGs 14, 15, and 18. Interestingly, we found that 96.82% of the alleles located within *pleio4.1* (source 4 in Figure 6B) had suppressive interaction with other alleles at the genome level (reparametrized coefficient < 0). Moreover, while PI-614889 alleles at *pleio4.1* (source 4 in Figure 6B) had a negative effect on DTF and PH and a positive effect on TKW, PI-614889 alleles at *pleio14.1* (source 14 in Figure 6B) had a positive effect on DTF and PH and a negative effect on TKW. Positive and negative SNP effects refer to the reparametrized coefficient (either < 0 or > 0) from the pairwise regression as described by Tyler et al. (2013).

### Identification of Putative Candidate Genes Controlling Agronomically Important Traits

We searched for candidate genes within the confidence intervals of all QTL. We reasoned that trait-related SNPs could be found within or close to the genes contributing to quantitative variation (quantitative trait genes, QTG). Altogether, 1,874 genes were found within non-overlapping confidence intervals of fifteen QTL (Supplementary Table 7). Nevertheless, we focused on the QTL *pleio4.1* and *pleio14.1* because of their pleiotropic effects on multiple traits and because they were common in F_2_ and F_3_ populations. Accordingly, the QTL *pleio4.1* and *pleio14.1* contributed to the phenotypic variation of three traits: DTF, PH, and TKW, and 282 genes were identified within their confidence intervals.

**FIGURE 7:**
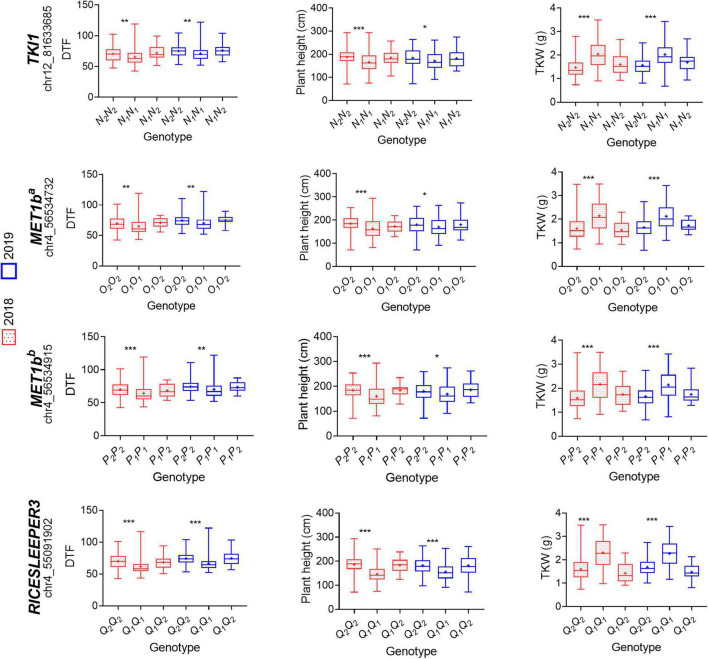
Evaluation of variant haplotypes using available whole-genome sequencing and phenotypic data of 310 quinoa accessions. Phenotypic effects of four haplotype variations within three candidate genes are shown: *TSL-KINASE INTERACTING PROTEIN 1* (*TKI1*) (SNP: chr12_81633685), *DNA (CYTOSINE-5)-METHYLTRANSFERASE 1* (*MET1b^a^*) (frameshift variant chr4_56534732), *MET1b^b^* (disruptive inframe deletion chr4_56534915), and *RICESLEEPER3* (disruptive_inframe_insertion chr4_55091902). The variants genotypes correspond to, for instance, *N_1_N_1_* (our homozygous parent PI-614889), *N_1_N_2_* (heterozygous), *N_2_N_2_* (our homozygous parent CHEN-109) and are described in Supplementary Table 4. Significant differences between genotypes are shown by asterisks (*t*-test, α < 0.05 = ***, α < 0.01 = **, α < 0.001 = *). Phenotypic data of different years are shown in different colors. DTF: days to flowering, TKW: thousand kernel weight.

Among the 282 genes described above, we found 41 genes with a previously described function related to flowering-time, photoperiod, and yield regulation in other plant species. Later, we compared the sequences of these genes between both parents of the population (Supplementary Table 8). From all the SNPs and InDels that differed between the parents for the selected genes, we chose those that were homozygous for each parent and had a putative effect on the function of the encoded protein. From 83 selected variants, we could only identify seven SNPs in the sequencing data of the F_2_ population. To assess the possible effects of the variants, we grouped the F_3_ plants according to the corresponding F_2_ genotype at the variant locus and performed t-tests (α = 0.05) to compare DTF, PH, and TKW between the groups. As result, none of the analyzed variants explained the phenotypic variance observed for DTF, PH, and TKW (Supplementary Figure 13). Afterward, we used available whole-genome sequencing and phenotypic data of a quinoa diversity set (310 quinoa accessions grown in a 2-year experiment in Kiel, Germany) (Patirange et al., 2020) to perform the same analysis. Namely, we grouped the diversity set based on the genotypes of the F_2_ parents (either CHEN-109 or PI-614889) at the variant locus and performed t-tests (α = 0.05) to compare DTF, PH, and TKW among the created groups. As result, we observed several significant phenotypic differences for PH and/or TKW and/or DTF when we grouped the quinoa diversity set based on the genotypes of our female and male parents (Supplementary Figure 14). Interestingly, while most of the investigated variants explained the phenotypic variation in one or two of the phenotypes (either PH, TKW, or DTF), only four variants within three genes explained the phenotypic variation of the three traits, simultaneously. These variants were: a missense SNP at *TSL-KINASE INTERACTING PROTEIN 1* (*TKI1*), a frameshift variant and a disruptive in-frame deletion at *DNA (CYTOSINE-5)-METHYLTRANSFERASE 1* (*MET1b*), and a disruptive in-frame insertion at *RICESLEEPER3* (Figure 7). Compellingly, *TKI1*, *MET1b* and *RICESLEEPER3*’s functions are related to growth alterations, flowering delay and pleiotropic effects in the model plant Arabidopsis (Roe et al., 1993; Kakutani et al., 1996; Soppe et al., 2000; Saze et al., 2003; Ehsan et al., 2004; Bundock and Hooykaas, 2005; Knip et al., 2012). In the quinoa diversity set, accessions that carried the PI-614889 genotype (*N_1_N_1_*) at the missense SNP of *TKI1* (chr12_81633685) flowered earlier, were shorter and showed higher TKW values than those carrying the CHEN-109 (*N_2_N_2_*) genotype. Accessions carrying the deletion and the frameshift variant in *MET1b* (CHEN-109 genotype) flowered later, were taller and had lower TKW values than those accessions without the deletion and the frameshift variant (PI-614889 genotype). A similar scenario was observed for the disruptive in-frame insertion at *RICESLEEPER3*, where the accessions carrying the insertion (CHEN-109 genotype) had higher values of DTF, PH, and lower TKW values (Figure 7).

## Discussion

We exploited the recent advances in sequencing technologies and computational analysis methods to localize QTL for agronomically important traits in quinoa. A high-density genetic map was constructed with a segregating F_2_ population, and 15 QTL were mapped with phenotypic data from two segregating generations. Candidate genes underlying the quantitative variation were identified within the QTL.

We calculated broad-sense heritabilities and genetic advance (GA) with a selection intensity of 5%, which resulted in moderate to high across all traits (excluding days to maturity). Interestingly, the high heritability coupled with high GA observed for days to flowering, panicle density, and saponin content indicates that selection may be more effective for these traits. Moreover, previous studies reported heritability values for days to flowering of 70.1% (2-year experiment with five quinoa genotypes) (Al-Naggar et al., 2017) and 91.0% (quinoa diversity set phenotyped for 2 years) (Patirange et al., 2020). The same studies calculated heritabilities of 89.7% for TKW and 68.0% for panicle density. Thus, the stated values in our study are in accordance with previous reports. Nevertheless, Al-Naggar et al. (2017) and Patirange et al. (2020) reported values of 85.0 and 60.7% for plant height. Differently, in our study, the traits plant height and panicle length showed moderate heritability, both with values of around 38%. A possible explanation is that the male parent showed a wide range and large variability in plant height, resulting in lower estimates of heritability.

Our study considered Skim-seq as a genome complexity-reduction method for constructing a genetic map for quinoa. In our hands, genotyping by Skim-seq was effective for QTL mapping and could be applied for a minor crop like quinoa, for which available resources and commercial interest are currently limited (Böndel and Schmid, 2021). We showed, by several quality controls, that the challenge of calling high-quality heterozygous SNP at low sequencing coverage (∼1.07×) could be overcome by modifications to conventional bioinformatic pipelines and imputation. Moreover, our results showed that whole-genome sequencing with coverage as low as ∼1.0× would be sufficient for QTL mapping. QTL mapping using whole-genome low-coverage sequencing has been successfully applied in chickpea and tobacco. In these studies, RILs and backcross populations were sequenced with depths from ca. 0.75 to 1.0×, and the number of markers for mapping ranged from ∼4,000 to ∼53,000 (Kale et al., 2015; Tong et al., 2021). Although Skim-seq was sufficient for constructing a high-density genetic map, there are limitations to this method. First, there are no tools available for the accurate imputation of InDels in F_2_ populations of polyploid crops. Thus, further uses of our genotypic data are restricted only to SNPs. The second important drawback of this approach is that the centromeric regions cannot be accurately sequenced due to their repetitive nature. However, these problems might be addressed soon by the development of more sophisticated bioinformatics tools for imputation (Jordan et al., 2022).

We found large gaps between adjacent markers in several linkage groups (up to ∼33 cM). Based on collinearity with the physical map, we determined that most of the gaps corresponded to centromeric regions (Figure 3). The genetic map of quinoa published by Jarvis et al. (2017), which served to produce the reference genome, is similar in this regard because it contained major gaps (up to ∼29 cM). It is possible that the existence of repetitive sequences in these regions complicate the identification of markers at these sites because of poor sequencing quality (Heitkam et al., 2020). Repeats have always presented technical challenges for short-read sequencing technologies (Zhao et al., 2021). Consequently, recombination fraction calculations would be affected by the lack of markers at the centromere, and fragmentation of chromosomes into two linkage groups could be expected. Furthermore, it has been suggested that the removal of highly distorted SNPs or bins during the construction of genetic maps is responsible for fragmentation of the chromosomes (Gong et al., 2020). This was also found in wheat, where a recent ultra-dense genetic map yielded 25 linkage groups for the 21 chromosomes of this crop (Langlands-Perry et al., 2021). Importantly, although the chromosomes in our linkage map are fragmented into subgroups, this does not restrict the use of our linkage map, whose quality was verified by several independent controls.

Using Skim-seq and phenotyping, we mapped 15 QTL for ten different traits with a wide range of explained phenotypic variation (from 0.43% for MS to 22.01% for PH, DTF, and TKW) and a wide range of the contribution of individual QTL to the total phenotypic variation (from 0.06 to 16.21%). QTL explaining the total phenotypic variance are unreachable because variation in quantitative traits is affected by many small-effect QTL (often undetectable) with additive and dominance effects, and QTL-QTL interactions. Importantly, in our study, the use of families in the F_3_ generation allowed plants of the same family to be considered as replicates, allowing measurement of environmental variances and thus, significantly increasing the power and precision of QTL detection. Accordingly, the accuracy of our QTL analysis is supported by the QTL *sap10.1*, found for saponin content. This QTL, which was previously reported in other studies (Jarvis et al., 2017; Patirange et al., 2020), harbors a *TRITERPENE SAPONIN BIOSYNTHESIS ACTIVATING REGULATOR 2* (*TSAR2*) basic helix–loop–helix (bHLH) transcription factor, which likely controls the production of anti-nutritional triterpenoid saponins in quinoa seeds. However, no QTL was found for saponin content in the F_3_ generation. This might be due to the method, which was used to phenotype saponin content in this generation. We bulked seeds from ten plants in the field corresponding to one F_3_ family. From these bulks, we took samples of 20 seeds for saponin analysis. Thus, bulked samples of 20 seeds might not have been true representatives of thousands of seeds from an F_3_ family. Moreover, the relatively low correlation coefficient between the saponin content measured in F_2_ and F_3_ generations might be also a result of the low efficiency of this method for measuring the saponin content in the F_3_ population. We suggest measuring the saponin content separately in multiple single plants of every F_3_ family to obtain more accurate values for this trait in F_3_.

We consider the common QTL among F_2_ and F_3_ populations, which depicted pleiotropy, as the most relevant QTL in our study. These QTL, explaining the phenotypic variation of days to flowering, plant height, and TKW, were located at Cq2B and Cq6B. In contrast, a putative pleiotropic locus controlling days to flowering, days to maturity, plant height, and panicle length was found at Cq2A by Patirange et al. (2020). The different outcomes might respond to the different type of population that was used in our study as compared to Patirange et al. (2020). Accordingly, we could expect different alleles segregating in our biallelic F_2_ population than the ones studied by Patirange et al. (2020), who used a quinoa diversity set comprising more than 300 accessions. Furthermore, we found a strong correlation between days to flowering, plant height, and TKW (Figure 1), which were the traits implicated in pleiotropy. This reinforces their QTL co-localization at Cq2B and Cq6B. Similar correlations, where quinoa plants flowering earlier are shorter and depict higher yield-related traits have been observed by Patirange et al. (2020) and Manjarres-Hernández et al. (2021).

We reported additive and dominance effects in a wide range of values, where the highest additive effect was observed for *pleio20.1* (-9.13), a QTL explaining the phenotypic variation of plant height, panicle length, and panicle density, and the highest dominant effect was reported for *pleio4.1* (8.74). However, these results solely show the effects of single loci. Since quantitative variation in phenotypes results from highly complex networks and epistasis, we aimed to investigate the allele effects (relative to the PI-614889 allele) at genome-wide level on days to flowering, plant height, and TKW. We observed that nearly half of the studied alleles had a minor simultaneous effect on these three phenotypes; thus, confirming the nature of these traits as quantitative. From this analysis, we could also corroborate that *pleio4.1* and *pleio14.1* are major QTL, which themselves explain 10.55 and 6.52% of the phenotypic variance, respectively. Moreover, while PI-614889 alleles at *pleio4.1* had a negative effect on DTF and PH and a positive effect on TKW, PI-614889 alleles at *pleio14.1* had a positive effect on DTF and PH and a negative effect on TKW. This is correlated with the additive effects calculated for the markers with the highest LOD score within each QTL (Table 4). The observed contrasting effects of PI-614889 alleles at *pleio4.1* and *pleio14.1* would probably complicate breeding processes, whose aim is to reduce days to flowering and plant height and increase TKW, simultaneously (Murphy et al., 2018; Patiranage et al., 2021). In a broader sense, our results from the genome-wide epistasis analysis revealed the complexity of the regulation of days to flowering, plant height, and TKW in quinoa, which was expected given the intricated processes, such as DNA methylation, histone modification, and non-coding RNA-associated gene silencing, which underlie these traits (Pandey et al., 2021).

A search for candidate genes within the confidence intervals of the QTL was performed. We reasoned that trait-related SNPs could be found within the genes contributing to quantitative variation. Within the 15 different QTL described in our study, we found homologs of known flowering time (*HEADING DATE 3A, WRKY TRANSCRIPTION FACTOR 13*, *FLOWERING LOCUS D*), plant architecture (*APETALA2-1*), and yield-related (*SMALL BASIC INTRINSIC 1-2, SWEET*) genes from other species (He et al., 2003; Yuan et al., 2014; Patil et al., 2015; Wang et al., 2015; Li et al., 2016; Ma et al., 2017). Besides, we identified *FLOWERING LOCUS T* (*CqFT2A*) within *pleio7.1*, a QTL found in the F_2_ population, exclusively. Although *FT* genes are described in studies related to flowering time regulation in quinoa and *C. rubrum*, no flowering-time function has been specified particularly for the *CqFT2A* paralog (Štorchová, 2020; Patiranage et al., 2021). To continue toward the identification of candidate genes, we mainly focused on the two pleiotropic QTL detected in F_2_ and F_3_ populations and identified putative candidate genes based on their known functions in flowering time and yield regulation in other species. Then, we investigated the effect of different sequence variants in these genes in a quinoa diversity set. As outcome and even though non-related accessions could have different haplotypes although they possess the same SNP or InDel in the candidate gene, we found several sequence variants that significantly explained the phenotypic variation of PH and/or TKW and/or DTF in the quinoa diversity set. Hence, the genes containing these variants (either SNP or InDel) might be interesting candidates for further studies. Among these genes, we found that sequence variation at *TSL-KINASE INTERACTING PROTEIN 1* (*TKI1*) had significant simultaneous effects on days to flowering, plant height, and TKW, which were the phenotypes whose variation was partly explained by the QTL *pleio14.1* in our study. In Arabidopsis, *TKI1* interacts with *TOUSLED* (*TSL*) and *TSL* loss of function mutations has pleiotropic effects on both leaf and flower development. Loss of *TSL* function also affects flowering time since it is required in the floral meristem for the correct initiation of floral organ primordia (Roe et al., 1993; Ehsan et al., 2004). Therefore, it is tempting to speculate that *TKI1* is involved in the regulation of the flowering time, flower development, and consequently, seed set in quinoa. Moreover, we found that sequence variations at *MET1b* and *RICESLEEPER3* have similar simultaneous effects on days to flowering, plant height, and TKW, as observed for the variant at *TKI1*. In Arabidopsis, *MET1* homozygous mutants displayed late-flowering phenotypes caused by ectopic expression of *FLOWERING WAGENINGEN* (*FWA)*, a regulator of flowering time. Hypomethylation, which correlates with the mentioned late-flowering phenotypes, is often accompanied by other developmental alterations (Kakutani et al., 1996; Soppe et al., 2000; Saze et al., 2003). Furthermore, *DAYSLEEPER. DAYSLEEPER*, the Arabidopsis homolog of *RICESLEEPER3*, encodes for a transposase-like protein essential for plant growth and development. Moreover, loss of function mutants of *DAYSLEEPER* showed retarded growth and delayed flowering (Bundock and Hooykaas, 2005; Knip et al., 2012). Importantly, the evidence of the role of these genes in the regulation of different biological processes is given for the model plant Arabidopsis while the observed pleiotropic regulation of days to flowering, plant height, and TKW in our study might be controlled by quinoa-specific genes. Hence, if *TKI1, MET1*, and *RICESLEEPER3* have the same function in quinoa can only be verified by further investigations. A first step toward elucidating the molecular mechanism governed by these genes might be expression analysis, for instance. Furthermore, haplotype analyses may focus on the up- and downstream regulatory regions of the most relevant candidate genes in our study. Besides, despite of the lack of reliable transformation protocols for quinoa, screening mutants and assessing their phenotypic effects seems to be another feasible approach for determining the function of these genes. Moreover, a recent study offers perspectives for functional studies in quinoa using virus-induced gene silencing (VIGS) (Ogata et al., 2021).

On the other hand, molecular markers linked to the pleiotropic QTL identified in the current study can be directly used in quinoa breeding programs for the simultaneous selection of different traits. Moreover, the provided information about QTL effects could guide breeders toward the selection of early, short, and high-yielding quinoa genotypes. Future work may address fine mapping of the interesting pleiotropic regions and characterization of candidate genes. Overall, the results presented in this study will help provide a framework for future research on the molecular mechanisms of flowering and other agronomically important traits and facilitate marker-assisted selection in quinoa breeding programs.

## Data Availability Statement

The datasets presented in this study can be found in online repositories. The names of the repository/repositories and accession number(s) can be found below: NCBI–PRJNA830312, SRR18906894 to SRR18907229.

## Author Contributions

CJ and NE directed the project and conceived to the research. NM-T designed the experiments, conducted the greenhouse experiment, carried out the bioinformatic analyses, and constructed the genetic map. NM-T and FB conducted the field experiment, performed the QTL mapping, and carried out the DNA isolation and marker analysis. KS carried out DNA sequencing and provided advice on subsequent bioinformatic analyses. NM-T together with all authors, wrote and finalized the manuscript.

## Conflict of Interest

FB was employed by Enza Zaden. The remaining authors declare that the research was conducted in the absence of any commercial or financial relationships that could be construed as a potential conflict of interest.

## Publisher’s Note

All claims expressed in this article are solely those of the authors and do not necessarily represent those of their affiliated organizations, or those of the publisher, the editors and the reviewers. Any product that may be evaluated in this article, or claim that may be made by its manufacturer, is not guaranteed or endorsed by the publisher.

## Footnotes

1
https://www.ncbi.nlm.nih.gov/bioproject/PRJNA830312
