# Supplementary material for: High-Density Mapping of Quantitative Trait Loci Controlling Agronomically Important Traits in Quinoa (Chenopodium quinoa Willd.)
Source: Front Plant Sci. 2022 Jun 9;13:916067. doi: 10.3389/fpls.2022.916067 (PMC9261497; doi:10.3389/fpls.2022.916067)
Supplement: Supplementary file 1 [file Data_Sheet_1.zip › Supplementary_Figures_and_tables.pdf]

Supplementary Material

1 Supplementary Figures and Tables

1.1 Supplementary Figures

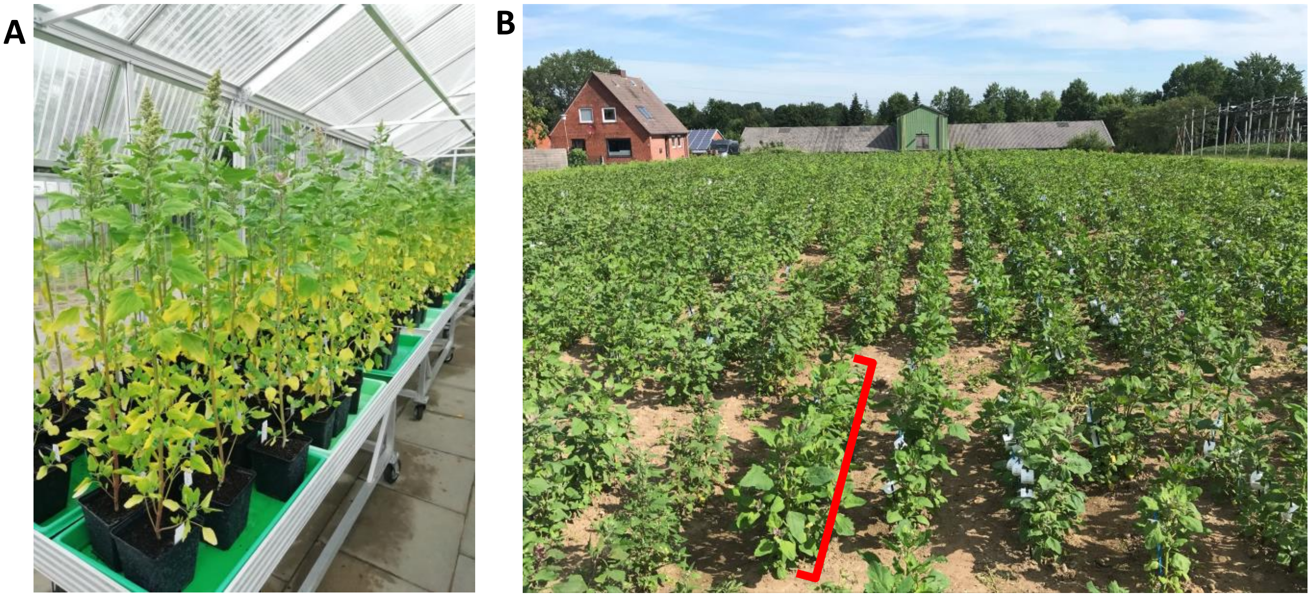

Supplementary Figure 1. Greenhouse and field experiments with F_2 and F_3 populations, respectively. (A) Eleven-weeks old F_2 plants in the greenhouse in 13x13x13 cm (width x length x height) pots. (B) F_3 families in a plant to row scheme nine weeks after sowing. One row representing one replicate of one F_3 family is shown by a square bracket.

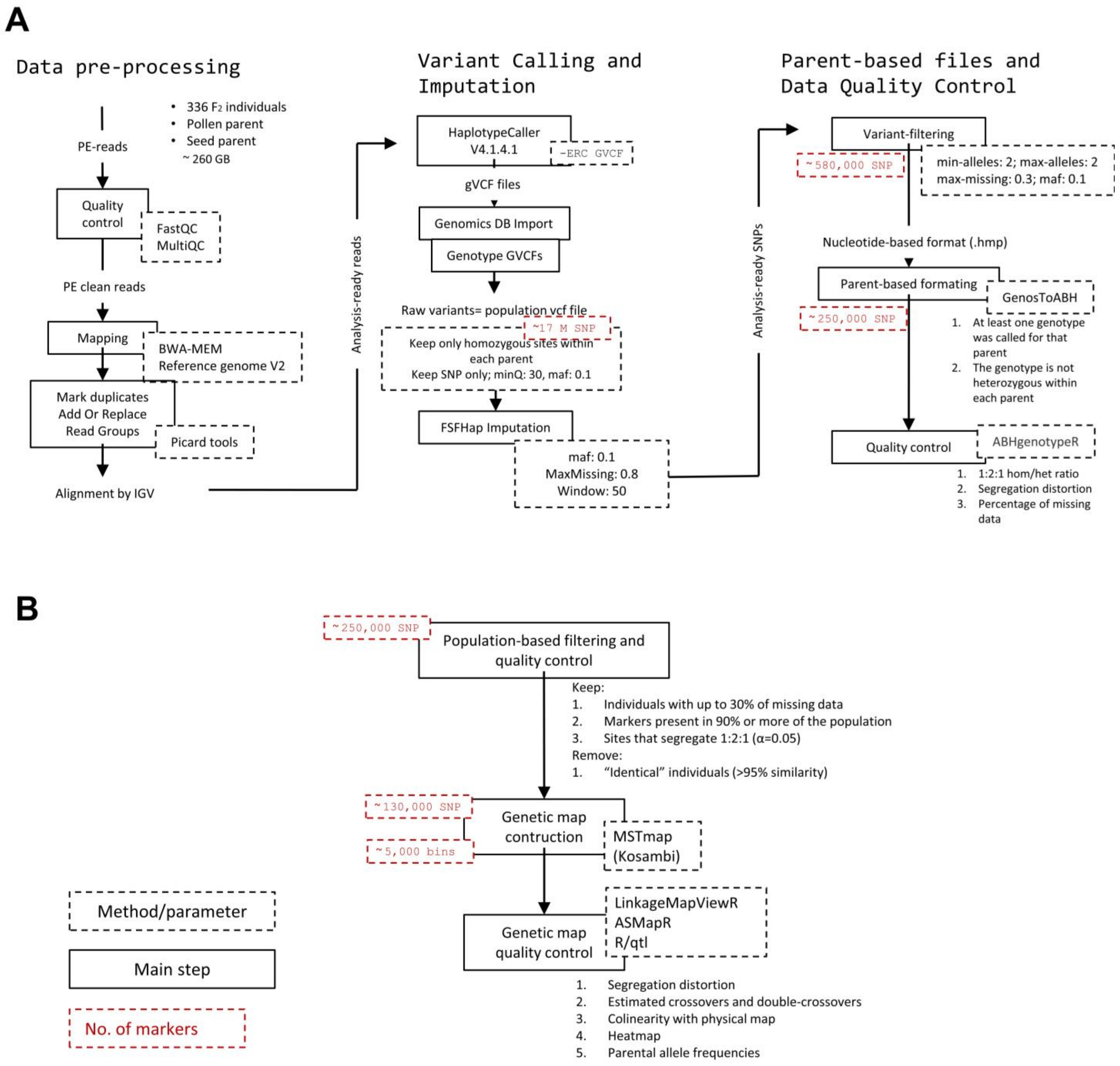

Supplementary Figure 2. Flow charts depicting the **(A)** bioinformatics pipeline and **(B)** the genetic map construction pipeline. Main steps of the pipelines are shown in solid-line boxes, method/parameters are shown in dashed boxes and the number of markers is shown in red dashed boxes. PE= pair end.

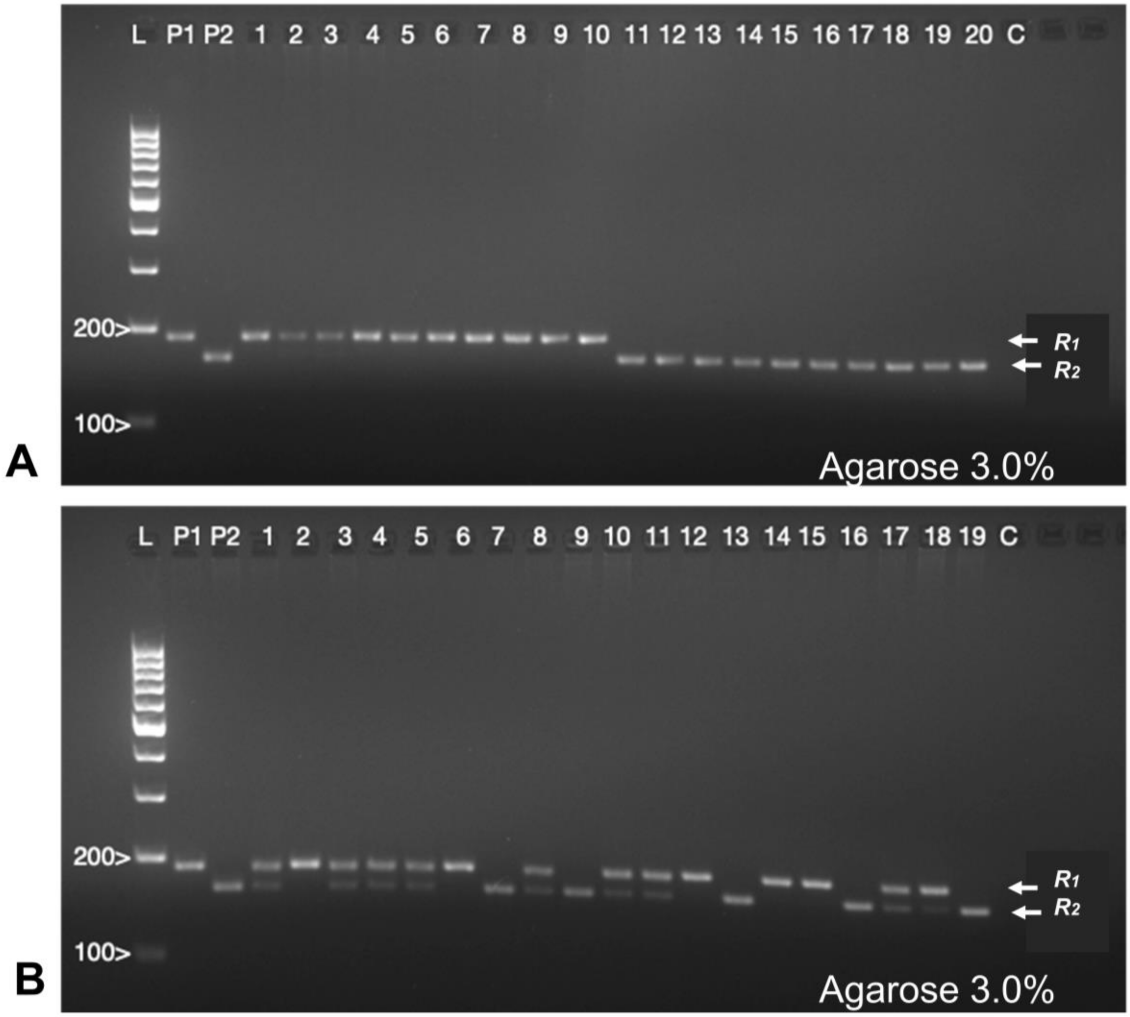

Supplementary Figure 3. Agarose gel electrophoresis of PCR products from the InDel marker JASS5. **(A)** Homozygous F_3 plants. From lane 1 to 10, plants are homozygous for allele R_1 (parent CHEN-109; 189 bp); and from lane 11 to 20, plants are homozygous for allele R_2 (parent PI-614889; 164 bp). **(B)** Plants from segregating F_3 families. L = middle range DNA ladder, P1 = parent CHEN-109, P2 = parent PI-614889, C = negative control. Agarose gels were run for 60 min at 100 V.

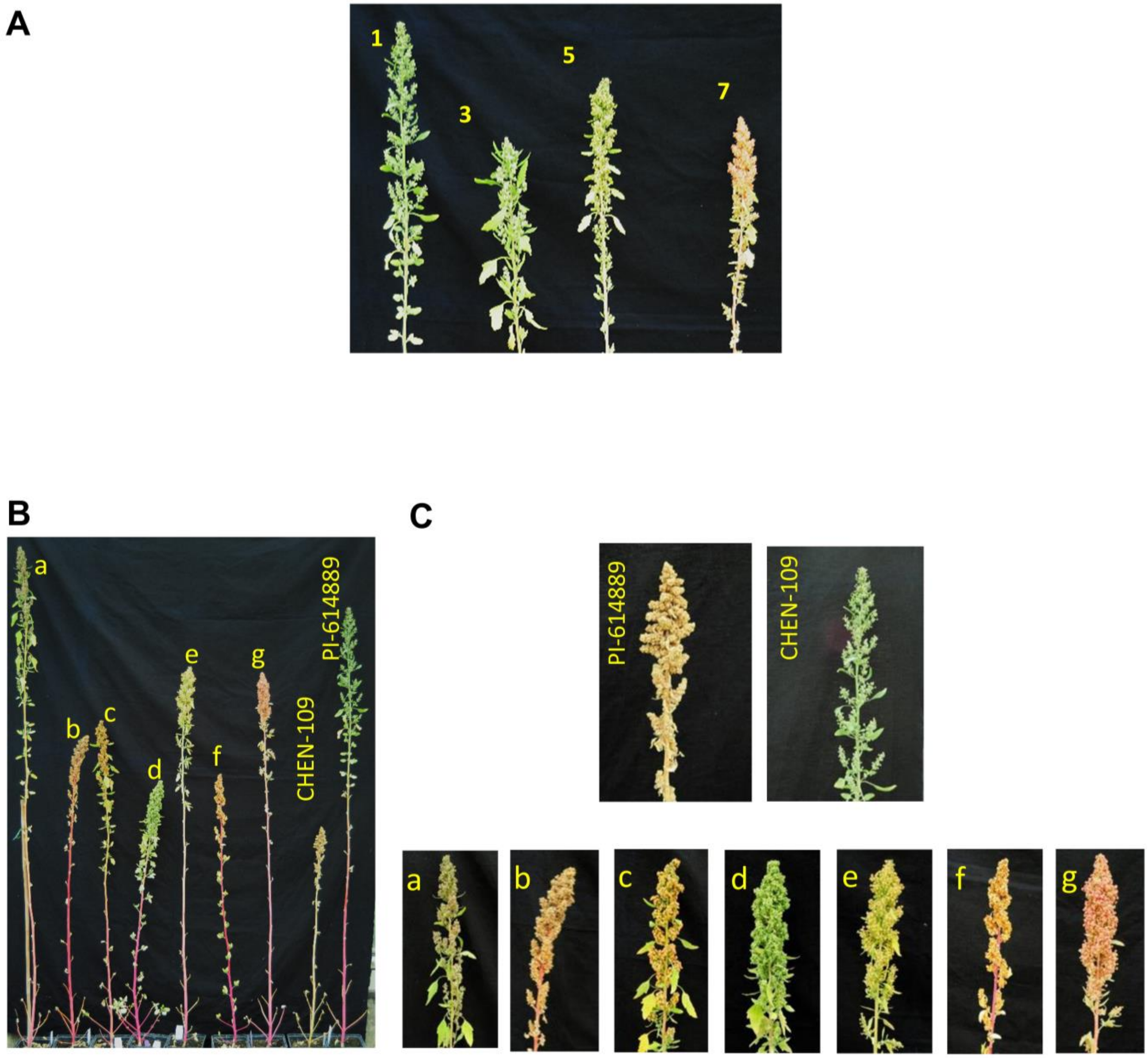

Supplementary Figure 4. Phenotypic variation in the F_2 population. **(A)** Variation of panicle density. Numbers in yellow represent the scoring scale used for phenotyping. **(B)** Variation of plant height illustrated by seven F_2 individuals (a to g) and the parental lines. **(C)** Variation in days to maturity illustrated by the different colors of the panicle. PI-614889 had reached maturity stage and was ready to harvest. CHEN-109 had not reach seed filling stage. Individual c is at seed filling stage. All pictures were taken 16 weeks after sowing.

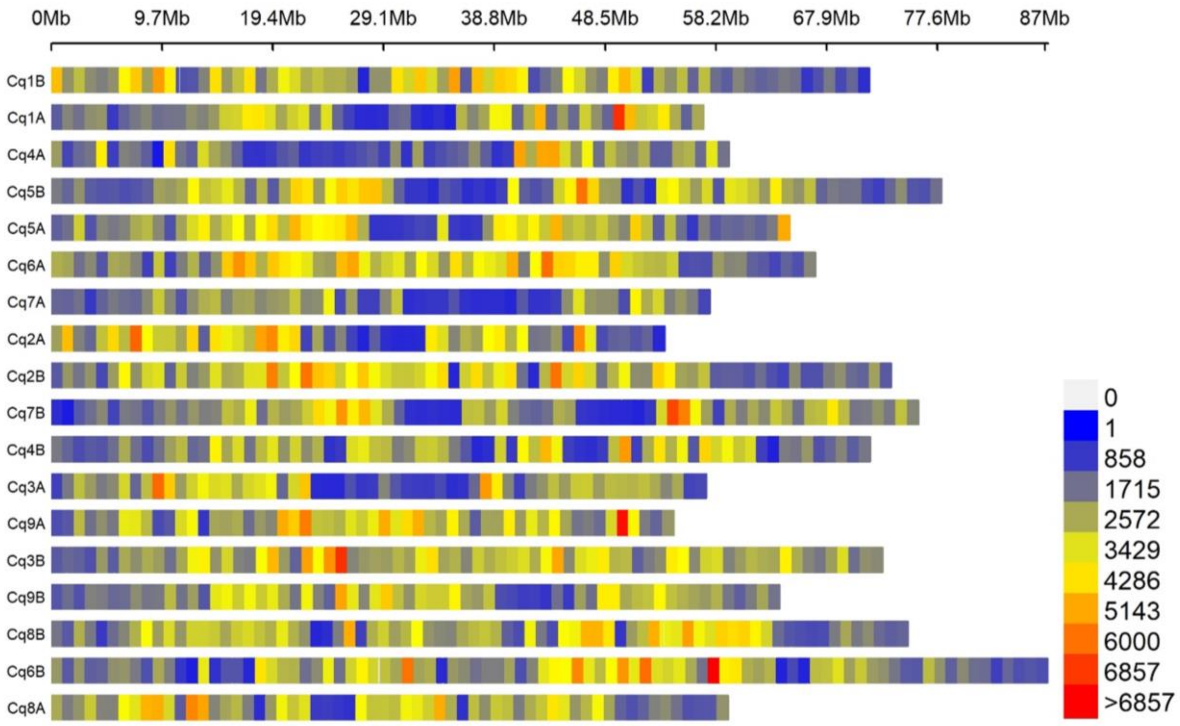

Supplementary Figure 5. SNP densities across 18 quinoa chromosomes. The number of SNP within 1 Mb window size are shown by different colors. Densities were calculated by CMplot R package using the raw data (~17 million SNP).

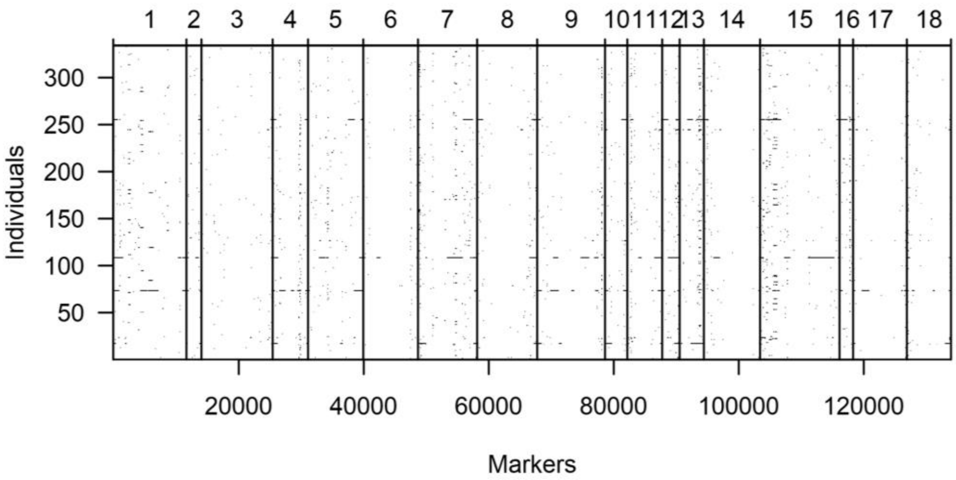

Supplementary Figure 6. Percentage of genome-wide missing data. Black dots show missing markers. Black horizontal lines represent several continuous missing markers. Chromosomes are separated by black vertical lines. Numbers above correspond to the quinoa chromosomes (even numbers: Cq1A to Cq9A; uneven numbers: Cq1B to Cq9B).

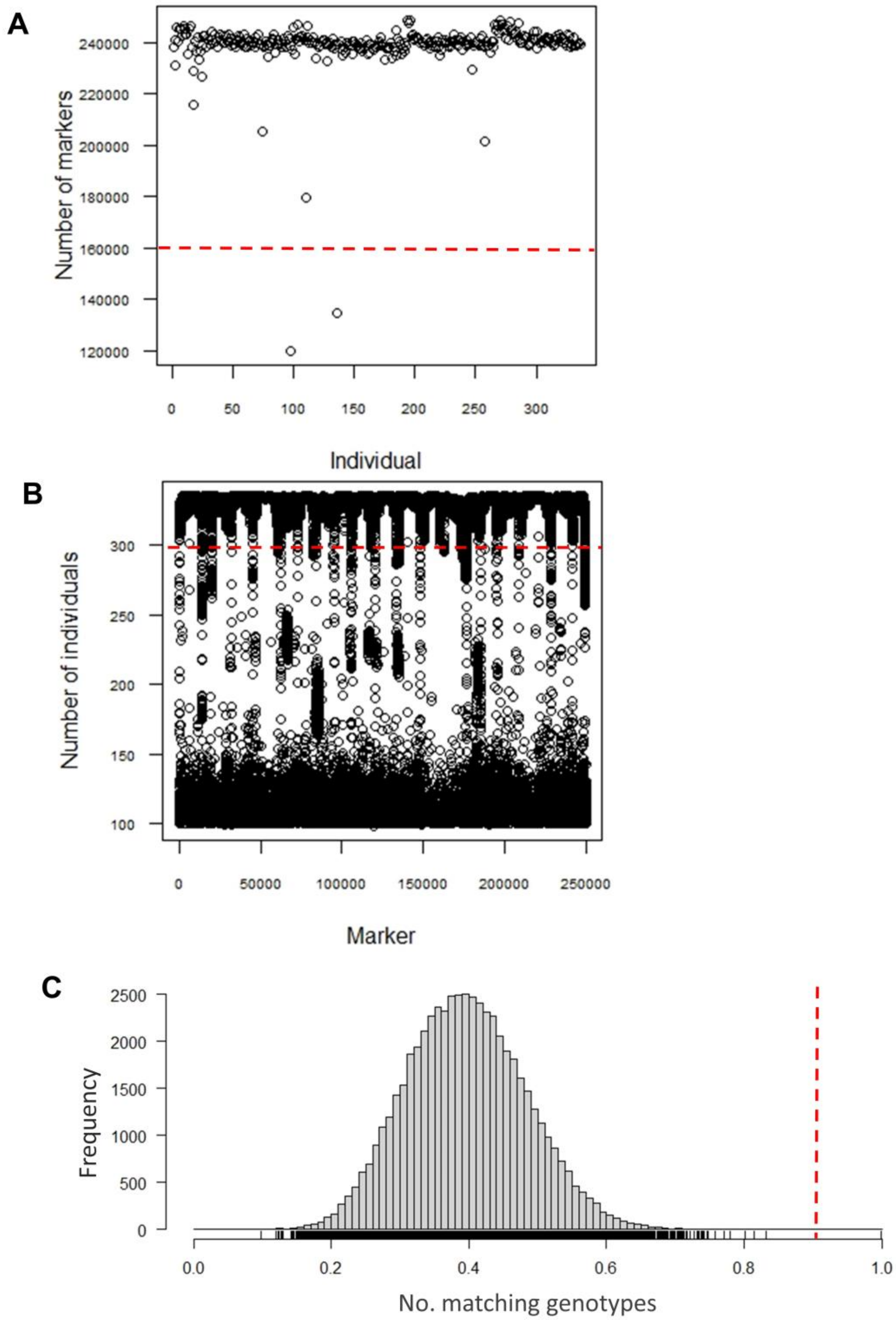

Supplementary Figure 7. Data filtering before map construction. **(A)** Number of markers per individual. **(B)** Number of genotyped individuals for each marker. **(C)** Histogram of the proportion of markers for which pairs of individuals have matching genotypes. Dashed red lines show the filtering thresholds. Data below the dashed red line in A and B, and to the right of the dashed line in C was removed.

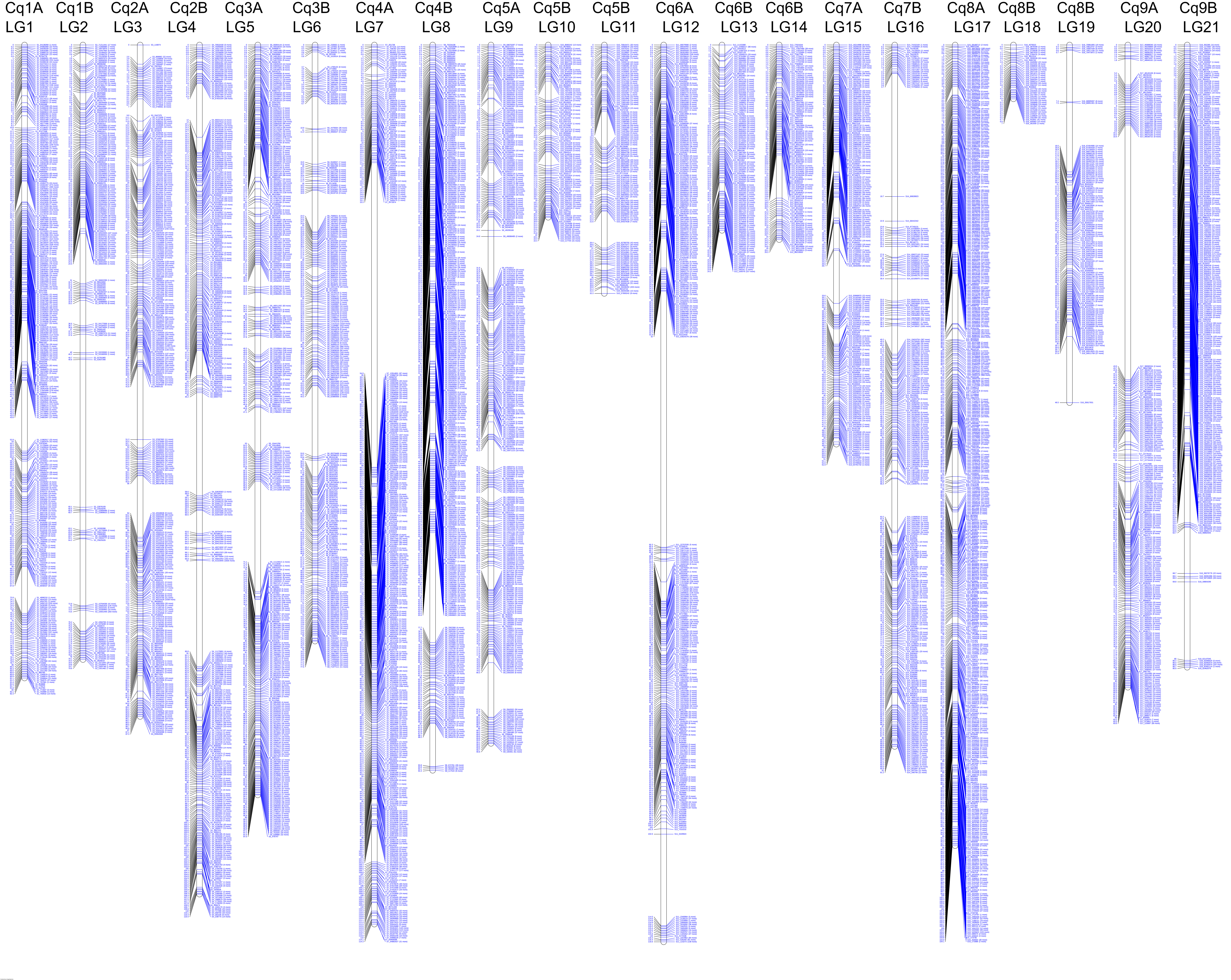
Cq1A LG1
Cq1B LG2
Cq2A LG3
Cq2B LG4
Cq3A LG5
Cq3B LG6
Cq4A LG7
Cq4B LG8
Cq5A LG9
Cq5B LG10
Cq5B LG11
Cq6A LG12
Cq6B LG13
Cq6B LG14
Cq7A LG15
Cq7B LG16
Cq8A LG17
Cq8B LG18
Cq8B LG19
Cq9A LG20
Cq9B LG21

Supplementary Figure 8. Quinoa linkage map based on 334 plants from an F_2 population derived from a cross between CHEN-109 and PI-614889. The map consists of 133,913 markers arranged in 5,218 bins and it was drawn with LinkageMapView R package. Numbers above indicate linkage groups (LGs) and chromosome numbers. Horizontal blue lines show the location of the first marker of each bin followed by the number of markers in each bin in parenthesis. Marker names are coded as “S” + Chromosome number + “_” + physical position of the marker. Genetic distances in cM are written to the left of each LG.

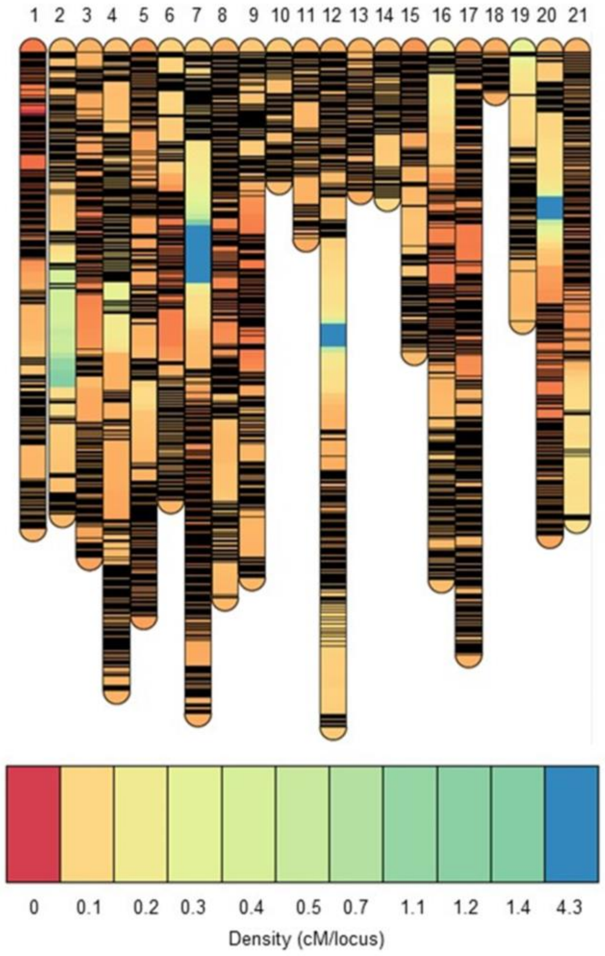

Supplementary Figure 9. Map density across 21 linkage groups from the F_2 population derived from a cross between CHEN-109 and PI-614889. Densities were recorded by LinkageMapView R package. Binned markers are shown by horizontal black lines.

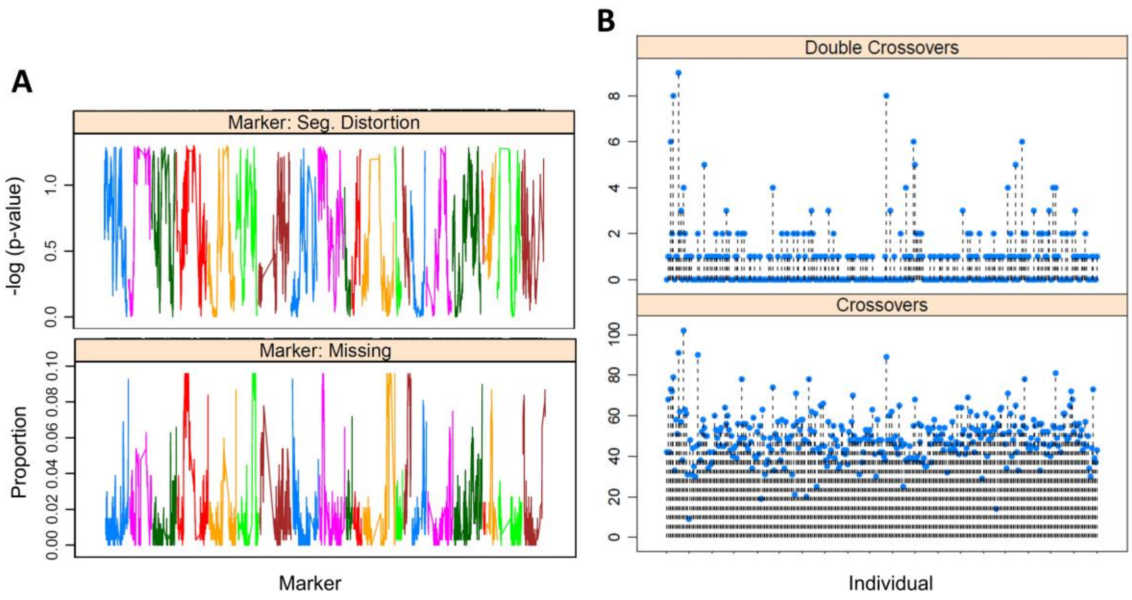

Supplementary Figure 10. Quality control of the marker data used for linkage map construction. **(A)** Marker's segregation distortion and missing proportion (number of individuals that are missing a marker in a specific locus / 334 individuals) calculated by ASMap R package; different colors correspond to each linkage group. **(B)** Estimated number of crossovers and double crossovers calculated by ASMap R package; blue dots represent the estimated values for each F_2 individual.

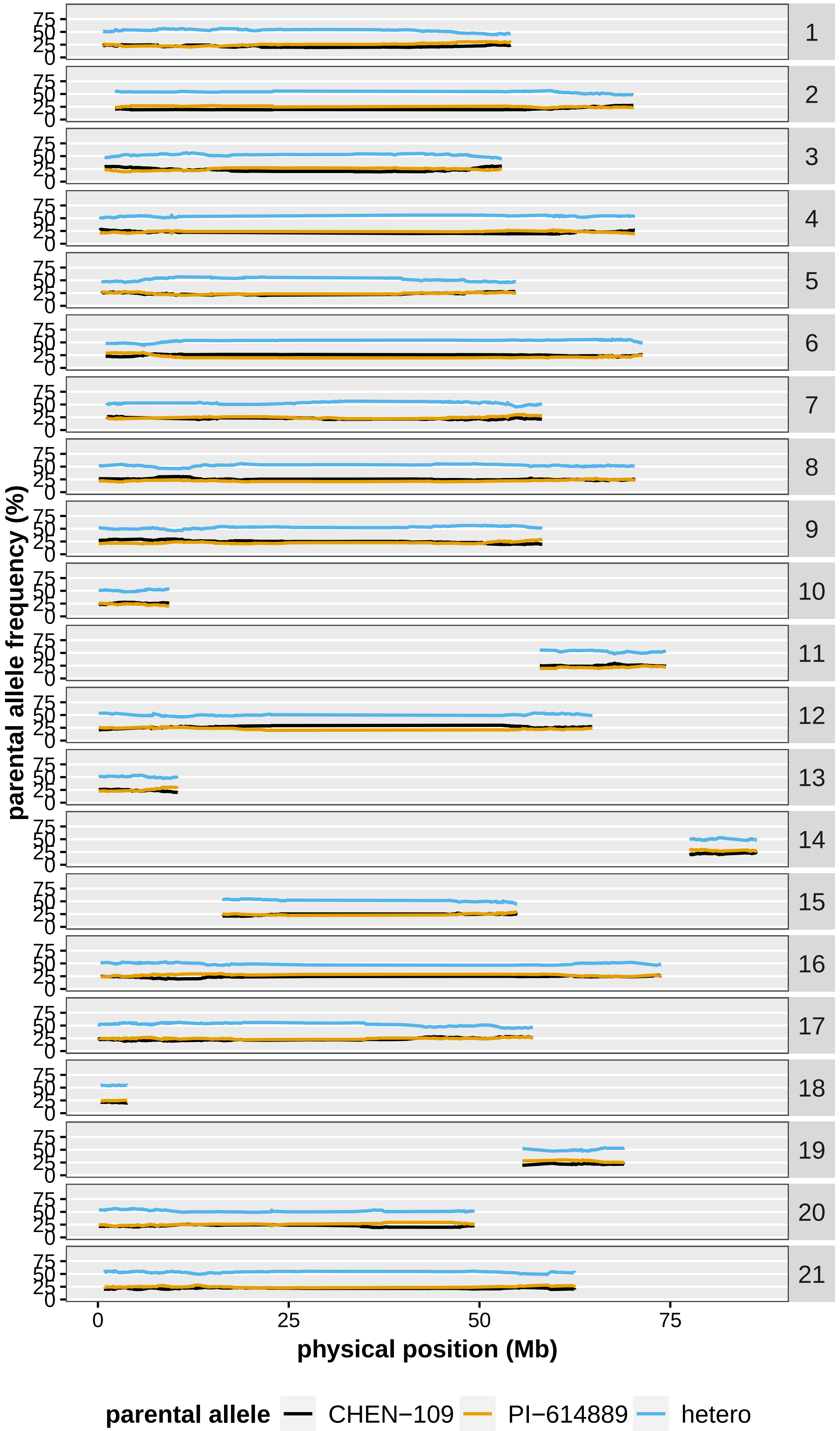

parental allele frequency (%)
0
25
50
75
1
2
3
4
5
6
7
8
9
10
11
12
13
14
15
16
17
18
19
20
21
0
25
50
75
physical position (Mb)
parental allele
CHEN−109
PI−614889
hetero

Supplementary Figure 11. Frequencies of the parental alleles calculated from the F_2 population using the program ABHgenotype R package in each of the linkage groups. Different alleles are shown by different colors. Linkage group numbers are shown to the right. The x axis shows the physical positions of the 133,913 markers according to the reference genome QQ74_V2. Hetero: heterozygous genotype.

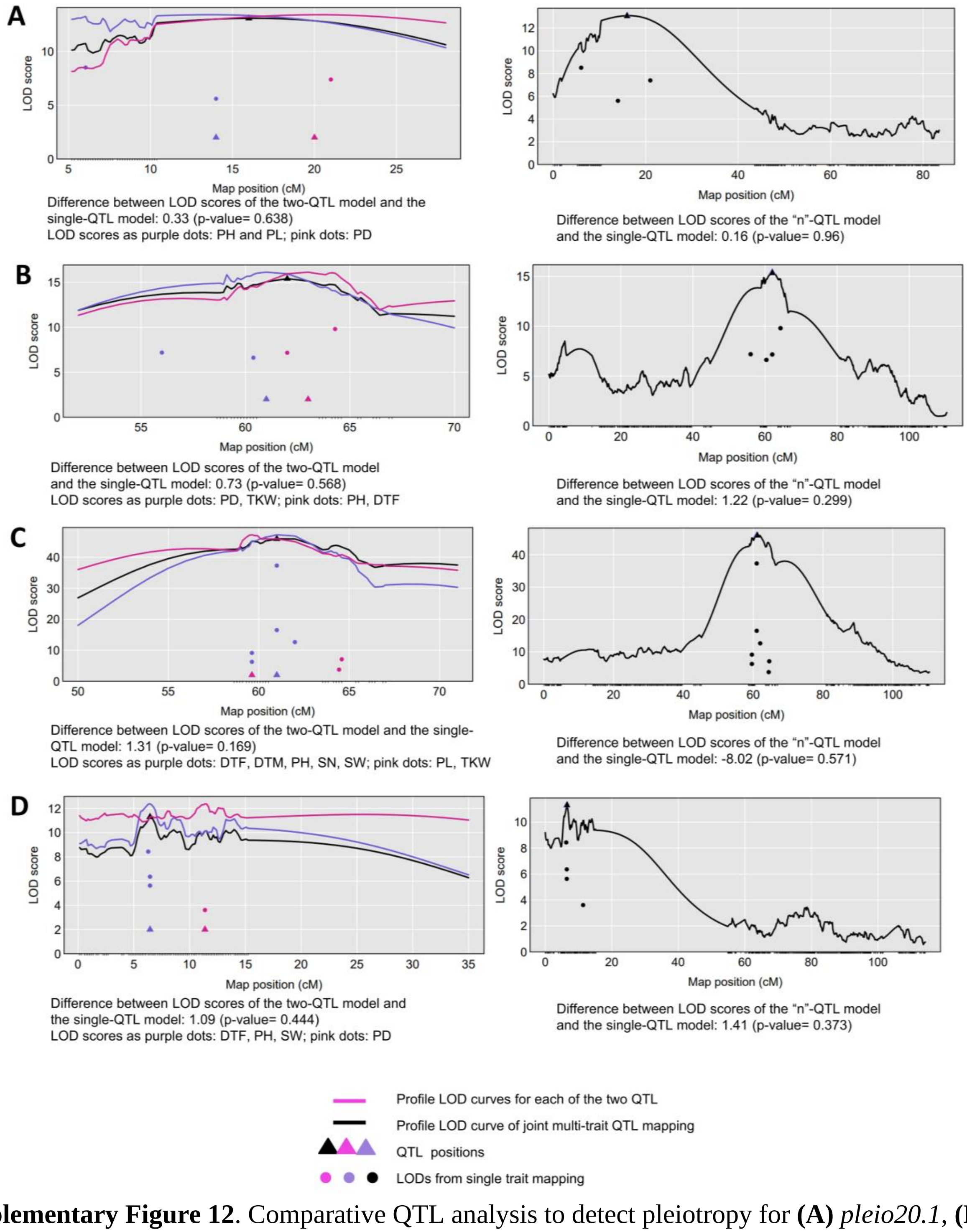

Supplementary Figure 12. Comparative QTL analysis to detect pleiotropy for **(A)** *pleio20.1*, **(B)** *pleio4.2*, **(C)** *pleio4.3* and **(D)** *pleio7.1*. Two tests were performed: one vs. two QTL (to the left) and one vs. “n” QTL (to the right). The black curve is the LOD score curve for the single-QTL model, with estimated QTL location indicated by a black triangle. The blue and pink curves are profile LOD score curves for the for the two-QTL model. Dots indicate the LOD score for the traits considering a single-QTL model. DTF: days to flowering, DTM: days to maturity, PH: plant height, PL: panicle length, PD: panicle density, SN: seed number per plant, SW: seed weight per plant, TKW: thousand kernel weight.

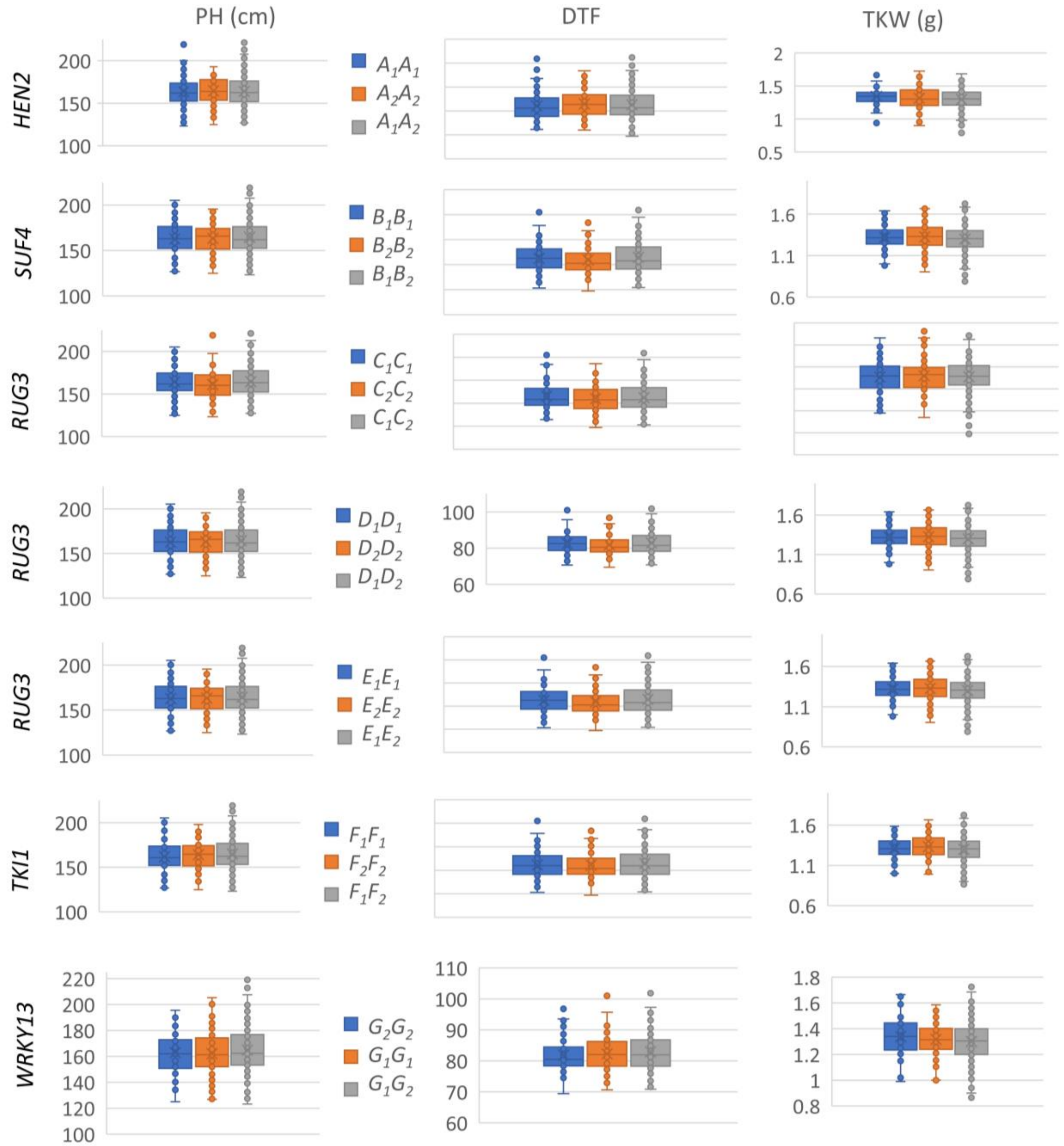

Supplementary Figure 13. Evaluation of variant haplotypes using the sequences of 328 F_2 individuals and the corresponding phenotypic data of 328 F_3 families. Phenotypic effects of haplotype variations within several candidate genes are shown: *DEXH-BOX ATP-DEPENDENT RNA HELICASE DEXH10* (*HEN2*), *SUPPRESSOR OF FRI 4 (SUF4)*, *RCC1 DOMAIN-CONTAINING PROTEIN 3* (*RUG3*), *WRKY TRANSCRIPTION FACTOR 13* (*WRKY13*) and *TSL-KINASE INTERACTING PROTEIN 1 (TKI1).* The variants genotypes correspond to, for instance, A_1A_1 (our homozygous parent PI-614889), A_1A_2 (heterozygous), A_2A_2 (our homozygous parent CHEN-109) and are described in Supplementary Table 4. Significant differences between genotypes are shown by asterisks (t-test, $\alpha<0.05$=***, $\alpha<0.01$=**, $\alpha<0.001$=***). DTF: days to flowering, TKW: thousand kernel weight, PH: plant height.

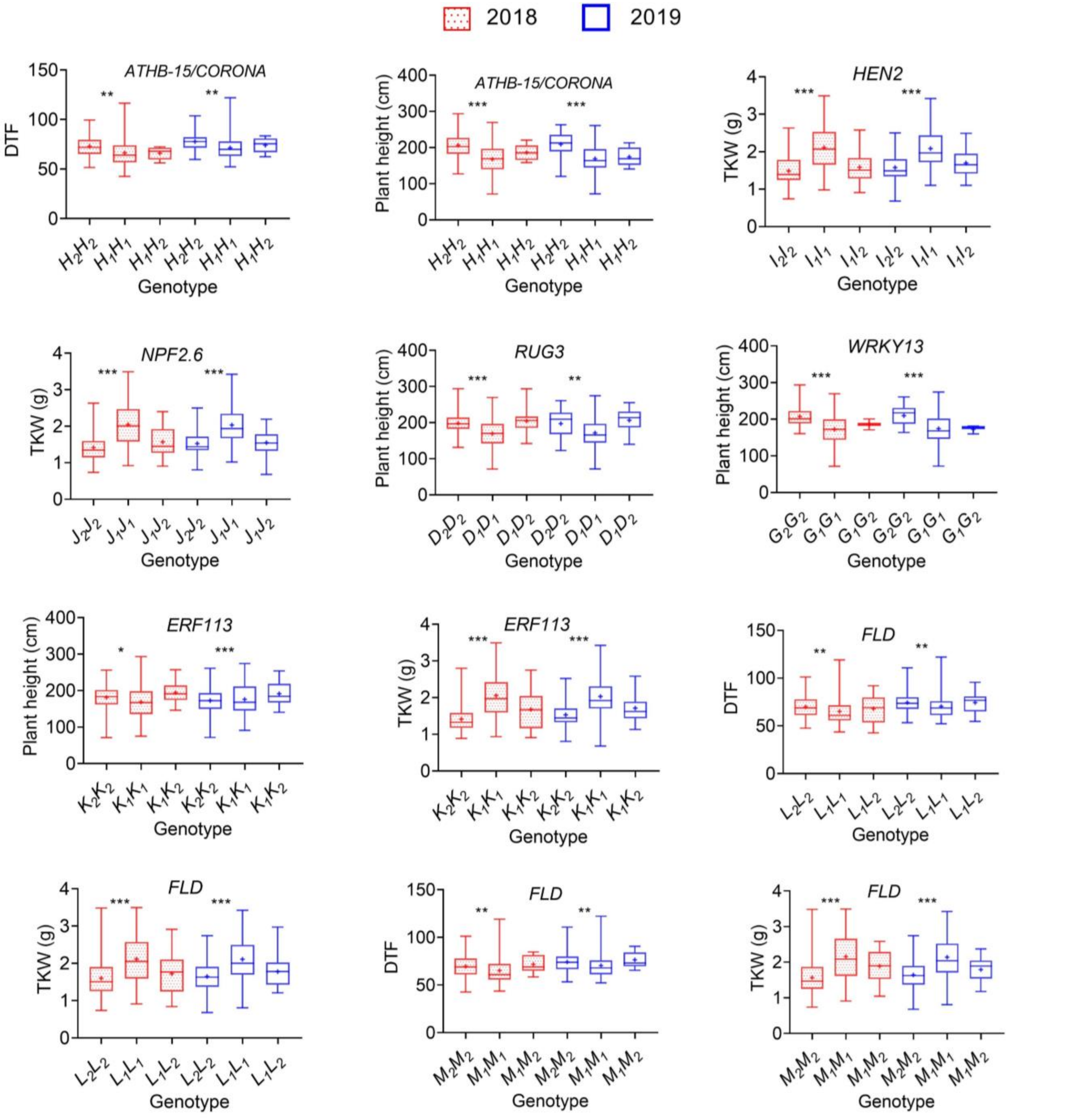

Supplementary Figure 14. Evaluation of variant haplotypes using available whole-genome sequencing and phenotypic data of 310 quinoa accessions. Phenotypic effects of haplotype variations within several candidate genes are shown: *ATHB-15/CORONA*, *DEXH-BOX ATP-DEPENDENT RNA HELICASE DEXH10* (*HEN2*), *NRT1/ PTR 2.6* (*NPF2.6*), *RCC1 DOMAIN-CONTAINING PROTEIN 3* (*RUG3*), *WRKY TRANSCRIPTION FACTOR 13* (*WRKY13*), *ETHYLENE-RESPONSIVE TRANSCRIPTION FACTOR 113* (*ERF113*) and *FLOWERING LOCUS D* (*FLD*). The variants genotypes correspond to, for instance, H_1H_1 (our homozygous parent PI-614889), H_1H_2 (heterozygous), H_2H_2 (our homozygous parent CHEN-109) and are described in Supplementary Table 4. Significant differences between genotypes are shown by asterisks (t-test, $\alpha<0.05$=***, $\alpha<0.01$=**, $\alpha<0.001$=***). Phenotypic data of different years are shown by different colors. DTF: days to flowering, TKW: thousand kernel weight.

1.2 Supplementary Tables

Supplementary Table 1. Plant material used in this study. DTF: days to flowering, DTM: days to maturity, PH: plant height, PL: panicle length, PD: panicle density, TKW: thousand kernel weight, SW: seed weight per plant, SN: seed number per plant, SC: Saponin content, MS: Mildew susceptibility.

Seed code	Generation	No. of plants/families	No. of plants genotyped	No. of plants phenotyped	
				Greenhouse	Field
171115	PI-614889 (female parent)	-	-	10	20
170876	CHEN-109 (male parent)	-	-	10	20 (DTM, SW and SN were not recorded)
190031	F_2	336	336	336 except for MS	-
191203-191562	F_3	334	-	-	DTF: 5,891<br>DTM: 2,343<br>PH: 5,860<br>PL: 5,860<br>PD: 5,860<br>MS: 6,346<br>SC: 330 families were bulked together (out of 334 sown families, four were lost due to biotic stress after germination)<br>TKW: 330 families were bulked together (out of 334 sown families, four were lost due to biotic stress after germination)<br>SW: 0<br>SN: 0

Supplementary Table 2. Methods for phenotypic evaluation.

Trait	Acronym	Description
Days to flowering	DTF	Number of days, from sowing until the first flower opens. Recorded thrice a week in the F_2 and twice a week in the F_3 population.
Days to maturity	DTM	Number of days, from sowing until the panicle is completely brown. Recorded twice a week.
Plant height	PH	Distance in cm from root collar to panicle apex, recorded once at BBCH-81 to BBCH-89.
Panicle length	PL	Distance in cm from panicle base to tip, recorded once at BBCH-81 to BBCH-89.
Panicle density	PD	Visual score based on the observed range of panicle density recorded at BBCH-81 to BBCH-89. The score ranges from 1 to 7: (1) “Low”: loose panicle with low number of spaced glomerules and panicle axes clearly visible; (3) “Intermediate”: high number of glomerules tightly arranged with panicle axis often visible; (5) “High”: high number of glomerules tightly packed and scarcely seen panicle axes; and (7) “Very high”: very high number of glomerules compactly arranged, panicle axes not visible. Recorded once at BBCH-81 to BBCH-89. An example of panicle density scoring in the F_2 population can be observed in Supplementary Figure 4.
Mildew susceptibility	MS	Performed only in the F_3 population (incidence only in the field). Performed as visual score ranging from 1 to 3: (1) “High”: symptoms observed in the whole plant or at least up to the upper third of the plant, (2) “Intermediate”: symptoms observed in the lowest two-thirds of the plant, (3) “Low”: no visible symptoms or only present in the lower third of the plant. Recorded once at BBCH-81 to BBCH-89.
Saponin content	SC	Recorded after harvest by foam test. Twenty seeds were placed in a 2 ml epi, shaken for 1 min at 1100 g in a GenoGrinder and incubated at room temperature for 5 min; later, the height of the formed foam (cm) was recorded (Jarvis et al., 2017).
Seed weight per plant	SW	Weight of the total harvest (whole panicle) per plant. Recorded once at BBCH-99.
Number of seeds per plant	SN	Number of seeds contained in the whole panicle per plant. Recorded once at BBCH-99.
Thousand Kernel Weight	TKW	Recorded after harvest.

Supplementary Table 3. Polymerase chain reaction (PCR) and agarose gel electrophoresis description.

<table>
<tr><td>Protocol/formulation</td><td colspan="2">Description</td></tr>
<tr><td rowspan="9">Master mix composition</td><td>Components</td><td>Volume (µl)</td></tr>
<tr><td>H_2O</td><td>15.9</td></tr>
<tr><td>PCR buffer (10x)</td><td>2.0</td></tr>
<tr><td>dNTP mix (10 Mm)</td><td>0.4</td></tr>
<tr><td>Forward primer (10 µM)</td><td>0.3</td></tr>
<tr><td>Reverse primer (10 µM)</td><td>0.3</td></tr>
<tr><td>Taq Polymerase (%U/µl)</td><td>0.1</td></tr>
<tr><td>DNA</td><td>1</td></tr>
<tr><td>Final volume</td><td>20</td></tr>
<tr><td>PCR amplification regime</td><td colspan="2">5 min at 94°C, 35x (30 s at 94°C, 30 s at 60°C, 60 s at 72°C) and 5 min at 72°C. The PCR was performed in a LifeTouch Thermal Cycler (Biozym Scientific GmbH, Hess. Oldendorf, Germany).</td></tr>
<tr><td>Agarose electrophoresis</td><td colspan="2">From each sample, 6 µl were mixed with 2 µl of loading buffer, and from the resulting mix, 2 µl were loaded for electrophoresis. Water was used as negative control. Gels (3.0% agarose) were run for one hour at 100 V and 400 Amp. After electrophoresis, the agarose gels were visualized in a UV transilluminator using a Bio-Rad Laboratories gel-imaging system. The images were analysed in order to determine the PCR products size by comparison with the DNA size ladder and to classify the samples according to their genotypic classes.</td></tr>
</table>

Supplementary Table 4. Allele and genotype nomenclature used in this study.

Gene name	Location	Allele name		Allele description		Variant type	Aminoacid change
		PI-614889	CHEN-109	PI-614889	CHEN-109		
HEN2	chr4_56438962	A_1	A_2	*A*	*T*	Intronic SNP	-
SUF4	chr12_80423460	B_1	B_2	*A*	*G*	3’ prime UTR SNP	-
RUG3	chr12_80551460	C_1	C_2	*C*	*T*	3’ prime UTR SNP	-
RUG3	chr12_80552842	D_1	D_2	*C*	*T*	Missense SNP	p.Ala388Thr
RUG3	chr12_80553379	E_1	E_2	*A*	*G*	Intronic SNP	-
TKI1	chr12_81633247	F_1	F_2	*A*	*T*	Intronic SNP	-
WRKY13	chr12_81728382	G_1	G_2	*C*	*A*	Missense SNP	p.Glu117Asp
ATHB-15/CORONA	chr4_51023634	H_1	H_2	*G*	*A*	Missense SNP	p.Asp22Asn
HEN2	chr4_56441565	I_1	I_2	*T*	*C*	Missense SNP	p.Ile724Met
NPF2.6	chr4_ 53557989	J_1	J_2	*C*	*T*	Missense SNP	p.Ala93Val
ERF113	chr12_81516821	K_1	K_2	*G*	*A*	Missense SNP	p.Pro326Leu
FLD	chr4_56844370	L_1	L_2	*A*	*AT*	3’ UTR InDel	-
FLD	chr4_56847562	M_1	M_2	*TGAAC*	*T*	Intronic InDel	-
TKI1	chr12_81633685	N_1	N_2	*T*	*A*	Missense SNP	p.Gln445Leu
MET1b	chr4_56534732	O_1	O_2	*ATT*	*A*	Frameshift	p.Gln222fs
MET1b	chr4_56534915	P_1	P_2	*AGTT*	*A*	Disruptive Inframe Deletion	p.Lys161_Leu162delinsMet
RICESLEEPER3	chr4_55091902	Q_1	Q_2	*A*	*AATTCCT*	Disruptive Inframe Insertion	p.Ile281_Thr282insProIle

Supplementary Table 5. Genetic and phenotypic segregation for two traits in the F_2 and F_3 populations. Red axil pigmentation was determined five weeks after sowing. The InDel marker JAASS5 was described by Zhang et al. (2017). R_1 and R_2 represent the 189 bp and 164 bp alleles, respectively.

Plants	**F_2 population**							**F_3 population**			
	Red axil pigmentation		$\chi^{2\,a}$	JASS5 genotype			$\chi^{2\,b}$	JASS5 genotype			$\chi^{2\,c}$
	Red	Green		R_1R_1	R_1R_2	R_2R_2		R_1R_1	R_1R_2	R_2R_2	
O	254	82	0.06	8	28	12	2.00	58	57	79	5.02
E	252	84		12	24	12		72.75	48.50	72.75	

E: expected, O: observed.
[a] 3:1 segregation, $\chi^2_{(0.95;1)} = 3.84$
[b] 1:2:1 segregation, $\chi^2_{(0.95;2)} = 5.99$
[c] 3:2:3 segregation, $\chi^2_{(0.95;2)} = 5.99$

Supplementary Table 6. Differences and concordance between original and masked-and-imputed genotypes in six different data sets.

Data set	Total SNPs	No. of masked SNPs	Missing data before imputation	Missing data after imputation	Differences between original and masked-and-imputed genotypes	Concordance between original and masked-and-imputed genotypes
Cq1A	191,864	8,179	91.0%	13.44%	0.31%	99.69%
Cq1B	289,447	982	94.8%	7.80%	0.07%	99.93%
Cq2A	204,038	3,925	88.7%	7.57%	0.25%	99.75%
Cq2B	317,962	1,948	83.7%	3.30%	0.05%	99.95%
Cq3A	200,718	2,260	87.3%	4.30%	0.08%	99.92%
Cq3B	288,428	6,187	74.1%	7.19%	0.14%	99.86%
